# Optic Flow: A History

**DOI:** 10.1177/20416695211055766

**Published:** 2021-12-06

**Authors:** Diederick C. Niehorster

**Affiliations:** Lund University Humanities Lab, 5193 Lund University, Lund, Sweden;; Department of Psychology, 5193 Lund University, Lund, Sweden

**Keywords:** optic flow, self-motion perception, Gibson, Grindley, Calvert, history, heading, aircraft landing

## Abstract

The concept of optic flow, a global pattern of visual motion that is both caused by and signals self-motion, is canonically ascribed to James Gibson's 1950 book “*The Perception of the Visual World.*” There have, however, been several other developments of this concept, chiefly by Gwilym Grindley and Edward Calvert. Based on rarely referenced scientific literature and archival research, this article describes the development of the concept of optic flow by the aforementioned authors and several others. The article furthermore presents the available evidence for interactions between these authors, focusing on whether parts of Gibson's proposal were derived from the work of Grindley or Calvert. While Grindley's work may have made Gibson aware of the geometrical facts of optic flow, Gibson's work is not derivative of Grindley's. It is furthermore shown that Gibson only learned of Calvert's work in 1956, almost a decade after Gibson first published his proposal. In conclusion, the development of the concept of optic flow presents an intriguing example of convergent thought in the progress of science.

## Introduction

**Figure 1.**
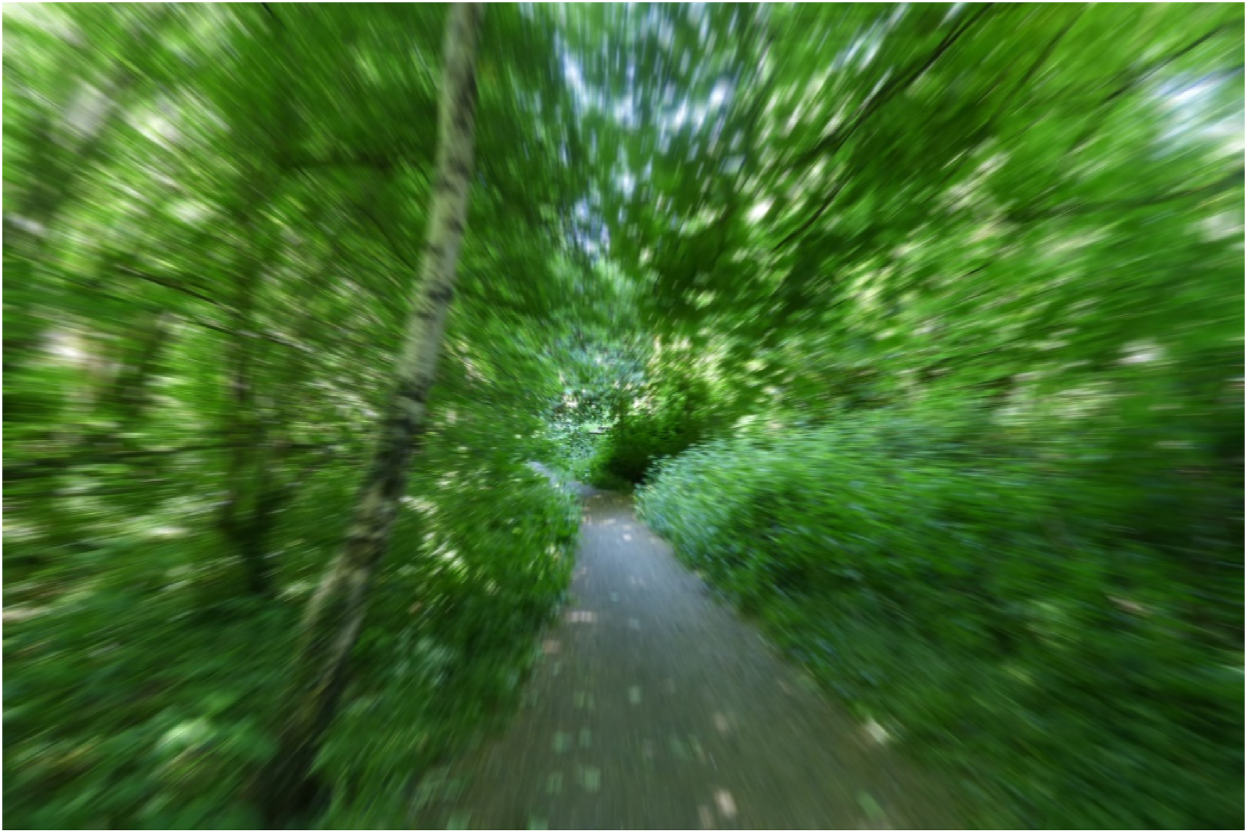
Impression of the radial optical motion experienced by an observer who translates along a wooded lane and does not rotate their eyes, head, or body. Adapted from “Wooded path to Weoley Castle from Selly Oak Park” [Photograph], by Elliot Brown, 2020, Flickr (https://www.flickr.com/photos/ell-r-brown/49934743043/). CC BY-NC-SA 2.0.

Imagine walking straight forward along a wooded path while looking straight ahead (Figure 1). When examining the pattern of light available at the eye as you move, note how the trees and shrubs appear to move outward in your visual image, becoming more and more peripheral as you approach and pass them. In fact, in your visual image, all these objects move radially away from an optically static point in the direction that you are heading toward. More generally, as the observer translates through the world or rotates, the pattern of light reflected from objects in the world to the point of observation undergoes a lawful continuous perspective transformation. These global motion patterns are called optic flow. Examples include the aforementioned radially expanding pattern across the visual field of the observer when moving forward, but also for instance, different kinds of lateral motion when translating or rotating sideways, and rotary or spiraling motion patterns when rotating along the line of sight.

Optic flow has several functions for behaving observers (see, e.g., Lee, 1980). For instance, observers can perceive their direction of self-motion from optic flow (Gibson, 1947, 1950; Warren, 1976; Warren et al., 1988; see Warren, 2008 for a review), and optic flow provides visual guidance for many actions (Warren, 1998, 2021) such as steering to a target (Calvert, 1954; Gibson, 1958; Warren et al., 2001), balance and posture control (Lee & Aronson, 1974; Stoffregen, 1985) and avoiding or achieving collisions (Fajen, 2008; Lee, 1976). Optic flow furthermore plays an important role in the perception of the shape of objects and the layout of the environment (Koenderink, 1986; Rogers, 2021; Todd, 1995).

In this paper, I discuss how the concept of optic flow was developed. The expanding flow of the visual world during translation could readily be noticed by any keen observer, especially since high-speed travel on trains, cars, and aircraft became widely available.^1^ Nonetheless, J.J. Gibson famously introduced it to perceptual psychologists (who, one would think, are keen observers) as late as 1947 after studying the visual guidance of aircraft landing (Gibson, 1947; see also Gibson, 1950). Perhaps unsurprisingly, Gibson was indeed not the only researcher to describe the flow of the retinal image during locomotion. As did Gibson, many of these other researchers described the phenomenon in the context of the landing of aircraft. In this paper, I will discuss the historical development of the concept of optic flow and review its treatment by each of its originators.

Besides Gibson, whose work is of course well known and often cited, optic flow and its role in vehicular guidance have also been described by the airfield lighting engineer E.S. Calvert. Calvert too receives a fair number of citations on his major papers (Calvert, 1954, 1957a; and the apparently lesser known, Calvert, 1950). As far as I am aware however, with the exception of Beall and Loomis (1996, 1997) who discussed Calvert's theory of visual control of aircraft, all these citations are tangential and no other text examines Calvert's contributions in detail.

The perceptual psychologist G. C. Grindley also discussed the role of visual motion in the landing of airplanes. However, before Mollon (1997) brought Grindley’s (1942) work, a secret report from the WWII era, to the attention of the vision science community, it was all but forgotten. Only a handful of texts cited it (to the best of my knowledge, these are Havron, 1962; Lybrand et al., 1959; Pitts, 1967, 1969; Schmidt, 1967; Taylor, 1988). It has been claimed (Mollon, 1997) that Gibson took the concept of optic flow from Grindley, without giving due credit. In this paper, I will discuss Grindley's report in detail and examine the claims made by Mollon (1997) regarding the influence of Grindley on Gibson. I also draw from other wartime reports by Grindley to show that Grindley himself built upon a theory devised by the psychoanalyst J.T. MacCurdy in the late 1920s considering the use of visual motion for aircraft landing.

This paper will extensively discuss the contributions of these four main players, Gibson, Calvert, Grindley, and MacCurdy, to the history of optic flow and discuss the extent to which each knew of the other, as uncovered through archival research. Sadly, until now the work of Grindley and Calvert remains mostly unknown, perhaps because it was for a good part detailed in secret reports. By means of this article, I aim to provide access to the contributions that Grindley and Calvert made to the development of optic flow. Furthermore, several other authors besides these main players have discussed the topic of optic flow in their work, but apparently not further pursued systematic treatments of it. These works will be discussed briefly in this article.

A fuller awareness of how the concept of optic flow evolved is not only of intrinsic interest, but also promotes a more complete understanding of optic flow and the many everyday percepts and behaviors it underlies. As with any concept, an appreciation of its historical baggage, of the challenges it survived during its evolution and the various missteps made during its development, sharpens one's grasp of the concept and may provide suggestive new ways of viewing it. As Herzberger (1966) states:

*Historical knowledge is important because it stimulates creative thinking. The man who first struggled with an idea, trying to find a law, looked at the situation with different eyes than do we who accept the law as a matter of course. He considered alternatives to the law, and different interpretations, and some of these alternatives and interpretations may still be stimulating and worth thinking about. (p. 1383)*

Besides, a more complete understanding of the scientific priority of an idea helps put its development not only in a scientific context, but also in context of its originators’ lives, providing one with a more complete view of the concept's antecedents. In view of this, in this article for each of the researchers who are part of the main storyline, I will place their treatment of optic flow in the context of their scientific life. I will also cite generously in this article because a lot of the relevant work can only be found in obscure reports whose existence is hard to find out about, which have otherwise been forgotten, or which are not known in the vision science community. The interested reader may contact the author for help acquiring these sources.

## Euclid

Besides the main characters in this story who are each discussed extensively below in sections “J.T. MacCurdy” to “E.S. Calvert,” several other renditions of optic flow, or ideas very relevant to optic flow, may be found in the literature. I will turn to these first. Note that I do not claim to provide an exhaustive overview.

Already the ancient Greeks, in the form of Euclid's *Optics* (3rd century before Christ), can be seen to have been acutely aware of the geometrical facts of not only static perspective, but also motion perspective^2^ (I thank Jan Koenderink for pointing this out to me, see also Theisen, 1984). Euclid's *Optics* contains theorems, with proofs and demonstrations, such as “When the eye moves nearer the object seen, the object will seem to grow larger” (Burton, 1945, p. 371), “Objects increased in size will seem to approach the eye” (p. 372), and “When the eye approaches the sphere, the part seen will be less, but will seem to be more”^3^ (p. 362). While in principle these theorems consider geometrical optics, it is noteworthy that they reference perception.^4^ Importantly, they present facts of the optical information available when relative motion between an observer and an object occurs, and as such these theorems have direct parallels with our current descriptions of optic flow. The reader is referred to Cutting (1986, pp. 25‒30) for further discussion of Euclid's *Optics* and its relevance to theories of perception.

## The Germans

Next, I turn to the German literature in which theoretical analyses or empirical findings are often found that predate and presage developments in the English literature (e.g., Todorović, 1996; Verstraten et al., 2015).

I have been hard-pressed to find a discussion in the German literature of the optical motions experienced during forward self-motion that refers to the expansion pattern (c.f., Figure 1), or how these motions may be used for the perception or control of self-motion. In this literature, discussions of the optical motions instead are usually limited to the observation that objects far away are seen to move more slowly than nearer objects, or some variation thereof involving distance from fixation, and are to the best of my knowledge invariably couched in terms of distance perception, not self-motion perception (see, e.g., Hell, 1981; Ono & Wade, 2005 for reviews).

Nonetheless, two sources were located that describe optical motion patterns experienced during forward self-motion in ways that are closer to our modern understanding of optic flow. Before discussing these, I first turn to a brief discussion of Helmholtz’ (1910/1925) contribution since his description of motion parallax is suggestive. Further related work investigating optokinetic responses to optic flow, such as the experience of vection in humans (Brandt et al., 1973; Fischer & Kornmüller, 1930; Mach, 1875/2001; Warren, 1895; see Riecke, 2011 for a review) will not be discussed here.

Helmholtz (1910/1925) has provided a famous description of motion parallax and how it makes relative depth relations clearly apparent:

*In walking along, the objects that are at rest by the wayside stay behind us; that is, they appear to glide past us in our field of view in the opposite direction to that in which we are advancing. More distant objects do the same way, only more slowly, while very remote bodies like the stars maintain their permanent positions in the field of view, provided the direction of the head and body keep in the same directions.^5^ Evidently, under these circumstances, the apparent angular velocities of objects in the field of view will be inversely proportional to their real distances away; and, consequently, safe conclusions can be drawn as to the real distance of the body from its apparent angular velocity. (p. 295)*

While Helmholtz in the above quote does not directly mention that objects at different sides of the observer appear to glide past in different directions, he quite possibly did not dwell on this part of his observation because of his focus on distance perception, for which the apparent speed would be primary. In my view, Helmholtz here nonetheless comes close to the classical description of the optic flow pattern experienced during forward self-motion.

At least two German publications predating Gibson may be seen to provide interesting discussions of the optical motions impingent on the observer during forward self-motion, their role in the control of self-motion and the perception of a stable world (Dauser, 1939; Tschermak-Seysenegg, 1939). Here I translate key sections of these two papers and provide a brief discussion. The original German versions of the translated sections are available in Appendix A. I thank Markus Lappe for helping to translate and interpret these German texts.

### Tschermak-Seysenegg (1939)

In a paper titled “On Parallactoscopy,” Tschermak-Seysenegg (1939) investigates depth perception during monocular viewing while making active or undergoing passive head movement relative to a configuration of objects and furthermore compares it to depth impressions derived from stereoscopic viewing. While in his experiments Tschermak-Seysenegg investigated perception during lateral body movements, he did provide a discussion of the visual motion impingent on the eye during forward self-motion. In the following quote, Tschermak-Seysenegg (1939) is seen to provide a description of the optical motion that makes clear reference to an expansion around the direction of self-motion:

*In the special case of approaching a set of objects (or when retreating from it!) which do not directly lie in the direction of travel, but instead are located to the right and left thereof, the set separates into individual sensations to the right and the left with differing escape velocities. This is especially the case when looking straight forward with respect to both sides of the driver's seat of a car or an airplane (at low altitude!) or when looking from the bow of a ship; the inverse is the case when looking from the stern of a ship or when looking out from the rear window of the viewing car of a train. In all of these cases, it is horizontal parallax that should be considered [the basis of] the depth impressions […]. (p. 456)*

As his reference to “velocities” in the above quote already suggests, it was clear to Tschermak-Seysenegg that visual motion, not just a change of perspective, is critical for depth impressions to arise. Specifically, later in the text, Tschermak-Seysenegg (1939) considers the depth impression available from two different consecutive views of a scene, but states that this does not provide the same kind of depth percept as is caused by visual motion:

*Such a “static” way of looking at things lacks the immediate and mandatory experience of depth which accompanies the apparent motion^6^ or the perception of motion. (p. 457)*

Lastly, Tschermak-Seysenegg (1939) discusses the role of motion parallax during fast, passive motion of an observer in another passage where he shows to have a clear understanding of the pattern of optical motion impingent on a moving observer:

*Special attention and evaluation is deserved for motion-based parallactoscopy in some flight maneuvers, especially when transitioning to land, i.e., already at low attitude, as tall objects (towers, chimneys, trees, telegraph poles, semaphores, power poles, houses, etc.) display a clear horizontal or specific vertical motion parallax with respect to the ground, and thereby allow a relative depth localization or estimate of height, but also an absolute judgment of distance. For aircraft, active head-body movements should be considered to be of at most secondary use. (p. 467)*

In sum, in this article Tschermak-Seysenegg is seen to keenly appreciate the optical motion patterns impingent on the eye during forward travel, and to understand that it is the visual motion per se, not the change of viewpoint, that causes the immediate depth percepts to arise. It is, however, noteworthy that although he does mention that the use of parallactoscopy for many activities, such as the steering of cars or even airplanes, should be tested (p. 468), his discussion concerned depth perception only. As such, Tschermak-Seysenegg at this time did not seem to appreciate the role these motion patterns may play for the perception or control of self-motion.

### Dauser (1939)

Dauser (1939) on the other hand, in a brief but insightful dissertation, is seen to discuss the use of motion patterns for the perception of self-motion, and for the perception of a stable, rigid world during self-motion. Despite this very general framing, in his dissertation Dauser (1939) only presents experiments investigating in which conditions stable objects in the world are perceived to move during head rotations at various rates; he did not examine perception during other types of self-motion, or the perception of self-motion.

Dauser (1939) starts with a discussion of the problem of relative motion, that is, the problem of whether the retinal shift of an object signals a change in object location, or self-motion. He says that classical theory would hold that this situation is resolved by making use of information about muscle movements of the observer. He then recognizes that during relative motion a second problem occurs: since everything that is depicted on the observer's retina undergoes perspective projection, during self-motion a perspective deformation of the image (“Bilddeformation,” Dauser, 1939, p. 5) occurs. Here again, he states that the deformation of the image alone does not allow determining if the object deformed or if the position of the observer's retina with respect to the object changed. Also, here, he states that an internal registration of the movements of the body should enable differentiation between the two cases. In connection to this discussion, Dauser (1939) then wonders:

*When the deformation of the image is due to movement of the body (head, eye), and not due to a change of the shape of the object, what is it then that we actually see? Do we see the objects and especially their shapes and contours unchanged and unmoved, and thus “objectively” as they are? Or, when we see the deformation-motion, is this perception already an illusion? […] When I, for example, move through my room along a straight line, perspective shifts will occur; and these could either be seen as movements of the objects, or not be seen due to a reinterpretation of retinal positions. (pp. 5–6, emphasis his)*

Dauser (1939) is seen here to struggle with the problem of whether the perspective deformation that occurs in the retinal image is perceived or not, because in some sense the motion and deformation of the object is seen (or at least registered by the visual system), yet the objects that are consciously perceived during self-motion appear to the observer as unchanged and static in the world. With “reinterpretation of retinal positions,” he likely meant that retinal shifts and deformations due to self-motion are discounted when interpreting the retinal motion signals, such as through some form of reference signal. Dauser's suggestion reminds one of the models of motion perception developed by Wertheim (1994, 2008). Wertheim's theory states that the perception of object motion or stationarity in space is the result of a comparison between the retinal velocity signals and a reference signal that encodes the velocity of the retinae in space. Importantly, the comparison between the retinal and reference signals is required for a percept of motion to arise, the retinal motion signal itself is not accessible. This model resolves the quandary Dauser is seen to struggle with: retinal motion of the world is required for the perception of a stable world during self-motion (a zero retinal signal during self-motion would yield the perception of a moving world), but is itself not perceived.

Later in his text, Dauser (1939) resolves his paradox of whether retinal motion is perceived or not by stating that the optical motion experienced during self-motion is both seen (unconsciously registered we would say) and not seen (consciously):

*[…] our normal perception encompasses the perspective shifts as illusory motions of rigid objects. Illusory perception of motions is thus the normal case; but — we do not experience them, we do not take them seriously. The concept of optical illusion is not suitable for describing this matter. We see something different from what it is, but we do not let ourselves be deceived*.

*But one must go even further. If we would not perceive the perspective shifts at all, then we would also not perceive our own change of location in object space. If we would on the other hand take the perspective shifts seriously, we would not identify any resting object as static anymore. Only the perception of the perspective shift together with the ignoring and the not-taking-seriously of this motion enables the perception of a stable world in which I am moving.^7^ The illusory motion is thus a constitutive element in the perception of a stable world in which the perceiver moves. (p. 16, emphasis his)*

This above quotes from Dauser (1939) make it clear that he was keenly aware of the patterns of retinal motion experienced during self-motion, calling them perspective shifts and deformations (c.f., Gibson, 1957a, see “The Motion Perspective Gradient” section below). Furthermore, he understood that these patterns of motion, that is, the perspective shifts themselves and not the constituent motion signals, are the stimulus for the simultaneous perception of a stable world and motion of oneself through it (c.f., also, Gibson, 1957a).

It is worth noting though that there is an important difference between Dauser's and Gibson's position. Dauser (1939) held that the case where a viewed object deformed and the observer is static cannot be distinguished from the case where the object is rigid and the positioning of the observer's retina with respect to the object changed. Non-visual information about motion of the observer would be needed to distinguish the two cases. Gibson (1957a) on the other hand stated “there is a possible basis in optical stimulation for the ability to distinguish between and among rigid, elastic, and multiple moving things. The basis lies in different mathematical modes of transformation and motion.”^8^ (p. 294) While Dauser (1939) is in principle correct, non-rigid deformations of objects that exactly match in their optical projection to rigid relative motion of the object are likely exceedingly rare.

## Fruitflies

Before turning to the main storyline, there is one further independent development to discuss, which regards early research into optomotor responses in insects (Kalmus, 1949; see also Borst et al., 2010; Srinivasan, 2011). Specifically, Kalmus (1949, see also his own summary of his work in a comment on Lee, 1980, pp. 178‒179) developed a mathematical description of optic flow, and presented a theory of how flies would respond to different optic flow patterns. While Kalmus (1949) does not contain a clear presentation of the optical patterns available during translatory or rotary self-motion,^9^ the paper is noteworthy for several reasons.

Firstly, Kalmus (1949) explicitly separated the optic flow pattern into a component due to rotation of the observer and a component due to translation. In his experiments, he presented either rotary or translatory optic flow and studied the fly's optomotor responses, finding that the fly turns with the direction of rotation of the pattern, but walks in the direction opposite to the translation suggested by the optical motion. The separation of optic flow stimuli into components due to translation and rotation is now commonly done when studying the perception of self-motion evoked by these stimuli (see, e.g., Li et al., 2009; Warren, 2008). For a description how to perform experiments on optomotor responses of insects to rotary stimulation at home with the kids, see Kalmus (1960, experiments 77 and 78).

Secondly, Kalmus (1949) described the stimulus that causes the optomotor response of his flies in terms of spatial integrals of the motion pattern, not in terms of the motion of individual points. He furthermore notes that it is ambiguous whether the optical motion of individual points is due to translation or rotation, but does not show that for the spatial integral over the hemisphere it in most cases is not. By means of this description, and his discussion of the experimental results, Kalmus (1949) shows a clear understanding that it is motion patterns (global motion) that drives the fly's optomotor responses. While his discussion of a neural mechanism able to determine the translation and rotation components in visual stimulation (p. 146) is suggestive, it is too general to say that it foreshadows the discovery of neurons with large receptive fields that are sensitive to global motion patterns (e.g., Saito et al., 1986; Wall & Smith, 2008).

Lastly, Kalmus (1949) makes an insightful comment regarding the problem of perceiving the motion of an object in the world when one is also moving. Remarkably similar to the contemporary flow parsing hypothesis (Layton & Niehorster, 2019; Niehorster & Li, 2017; Rushton et al., 2018; Rushton & Warren, 2005; Warren & Rushton, 2009), Kalmus (1949) proposes that:

*one's brain must make a comparison between the observed relative motion of the objects in the field of view with a prediction of the relative motion of the objects in the field of view due to parallax. (p. 146)*

## J.T. MacCurdy

Surprisingly, the main story in this paper starts with a psychoanalyst, the Canadian-born John Thomson MacCurdy (*1886–†1947). After gaining a Bachelor of Arts in Biology in 1906 from the University of Toronto and proceeding an MD from Johns Hopkins University in 1911, MacCurdy worked for a time at Alzheimer's laboratory in Munich (Banister & Zangwill, 1949). Here, his interests were swayed to psychoanalysis and during this time he also met Sigmund Freud, whose method of psychoanalysis he would endorse for the rest of the decade (Forrester, 2008), and Carl Jung. In 1913, he returned to the USA to take up a lectureship in medical psychology at Cornell. Simultaneously, he took up a position as assistant to August Hoch, director of the Psychiatric Institute in New York, for whom he would edit a posthumous book (Hoch, 1921), which he would later build on for his theory of emotion (Banister & Zangwill, 1949; MacCurdy, 1925). As the USA entered the First World War, MacCurdy had already written a short book about the psychology of war (MacCurdy, 1917). Perhaps because of this preparation, he became an assistant to the army psychiatrist Thomas Salmon and was sent to England by the American Expeditionary Forces to prepare for the problems of war that were about to confront American army psychiatrists (see MacCurdy, 1918, for a book based on his experiences in the war).

The separation from his wife Winifred in 1922 was the immediate cause for a new chapter in MacCurdy's life; his coming to Cambridge, England in 1923 to take up a lectureship in Psychopathology (Bury, 1952; as cited in Forrester, 2008). At almost the same time, MacCurdy published his book *Problems in Dynamic Psychology* (MacCurdy, 1922), in which he broke with his years of support of Freudian psychoanalysis, much to the annoyance of Freud (Forrester, 2008). Leaving behind clinical practice, in Cambridge MacCurdy turned to a life as a teacher and forceful commenter on contemporary developments (Banister & Zangwill, 1949; Forrester, 2008). For instance, in his book *Mind and Money*, MacCurdy (1932) cast economics as a problem of applied psychology (“Obituary: John Thomson MacCurdy, M.A., M.D., Sc.D,” 1947), while another book (MacCurdy, 1944) was in bulk concerned with an analysis of Russian society.

MacCurdy also took on engagements outside Cambridge. Soon after his move to England, he became a consultant psychologist with the Royal Air Force (RAF). In this capacity, he visited Iraq and the Middle East to study flying conditions over the desert (Banister & Zangwill, 1949) and advised on the scientific selection and training of flying personnel (the lectures he gave on this topic to RAF personnel during the Second World War were the basis of a book, MacCurdy, 1943). During the Second World War, MacCurdy played an important role in producing black propaganda for the Political Warfare Executive unit of the Foreign Office that aimed to incite desertion and self-infliction of non-fatal illnesses among German troops (Richards, 2010). Also during this time, in a secret report, MacCurdy analyzed one of Hitler's speeches to inquire into his state of mind (MacCurdy, 1942).

From MacCurdy (1934), we learn that he has studied the technique of flying since the late 1920s. Although no other publications came from this effort during his longstanding association with the RAF, MacCurdy's study of flying technique is where I start this story of the history of optic flow.

Sadly, I have not been able to track down any writing by MacCurdy in which he discusses his theory of the use of visual information in the landing of aircraft. However, we know of MacCurdy's work from reports written by a fellow Cantabrigian, G.C. Grindley (see the “G.C. Grindley” section). Grindley first mentioned MacCurdy's theory of aircraft landing in a meeting with several RAF commissioned officers (recorded in Proposed psychological research, 1940) with whom he worked for the RAF's Flying Personnel Research Committee (FRPC). In a later progress report to the FPRC, Grindley (1940) clearly albeit briefly described the theory. In lieu of an original source upon which to base a discussion of MacCurdy's theory, below I reproduce in full Grindley’s (1940) short discussion of MacCurdy's theory of aircraft landing (underline his):

*Dr. J.T. MacCurdy, more than ten years ago, put forward a theory of landing which stressed the importance of the relative movements of objects in the pilot's visual field. If, for instance, the machine is approaching the ground in a straight line, if the attitude of the machine remains constant, and if the pilot keeps his eyes stationary in relation to the machine, the point on the ground which is being approached will give a stationary image on his retina (the “zero point”), while all other objects on the ground will give images which diverge from this point at speeds which depend on their distance and direction from the zero point and upon the height, air speed, and angle of descent of the machine. The important thing about this is that the pattern of movements on the pilot's retina (if the pilot can respond to it) does give an accurate indication of height, direction, speed, etc. which is independent of changes in visibility or in the nature of the landing ground. When (as happens in an actual landing) the path of the machine through the air and the attitude of the machine are both varying continuously, calculation of the retinal stimulation involves a number of complex geometrical problems; which I am now trying to work out and to present in an easily intelligible form*.

*In regard to the possible applications of MacCurdy's theory to flying training, I have made a few observations (on still days). In these the instructor landed the machine repeatedly (keeping in touch with me through the speaking tubes), while I observed the visual phenomena. I formed the impression that in a day-light approach phenomena such as the zero point can be observed when there is nothing else to attend to, but that the very natural tendency to perceive the aerodrome etc. as things of constant size and shape made more difficult the appreciation of the actual pattern of retinal stimulation. In spite of this it seems possible that it could be advantageous, from the start, to direct the pupil's attention to those visual stimuli (e.g. visual movements) which can be relied upon as guides to an accurate landing. In the case of night landings phenomena such as the apparent constancy of size of an aerodrome are likely to be less important, and it seems even more likely that a training in the visual movements to be expected would be useful*.

*I hope to submit, shortly, a report on this problem and on the possible applications of MacCurdy's theory. (pp. 6–7)*

Grindley submitted his report on the use of visual information in aircraft landing to the FPRC in 1942, although he failed to mention MacCurdy as the source of the theory. I will discuss Grindley's work next.

## G.C. Grindley

Gwilym Cuthbert Grindley (*1903–†1976, Figure 2), addressed as “C” by those who knew him (Mollon, 1997; Zangwill, 1977), first worked in physics at Bristol University as a research assistant to Arthur Mannering Tyndall (Grindley & Tyndall, 1924; Tyndall et al., 1928; Tyndall & Grindley, 1924, 1926a, 1926b). Grindley had, however, already decided to move to psychology (Zangwill, 1977) and was supported in this by the British comparative psychologist Conwy Lloyd Morgan (Davis, 1987), an early champion of learning and the animal mind (Costall, 1998; Grindley, 1936). Consequently, Grindley's first psychological work was in animal learning (Grindley, 1927a, 1927b, 1929, 1937) and included studies with another supporter of his move, the retired physicist-turned-chicken farmer Arthur Prince Chattock (Chattock & Grindley, 1931, 1933). Among Grindley's early work was also the first rigorously controlled laboratory demonstration of instrumental conditioning (Davis, 1987; Grindley, 1932). Grindley would later come back to the topic of learning in human participants (Dees & Grindley, 1951; Elwell & Grindley, 1938; Macpherson et al., 1948, 1949).

**Figure 2.**
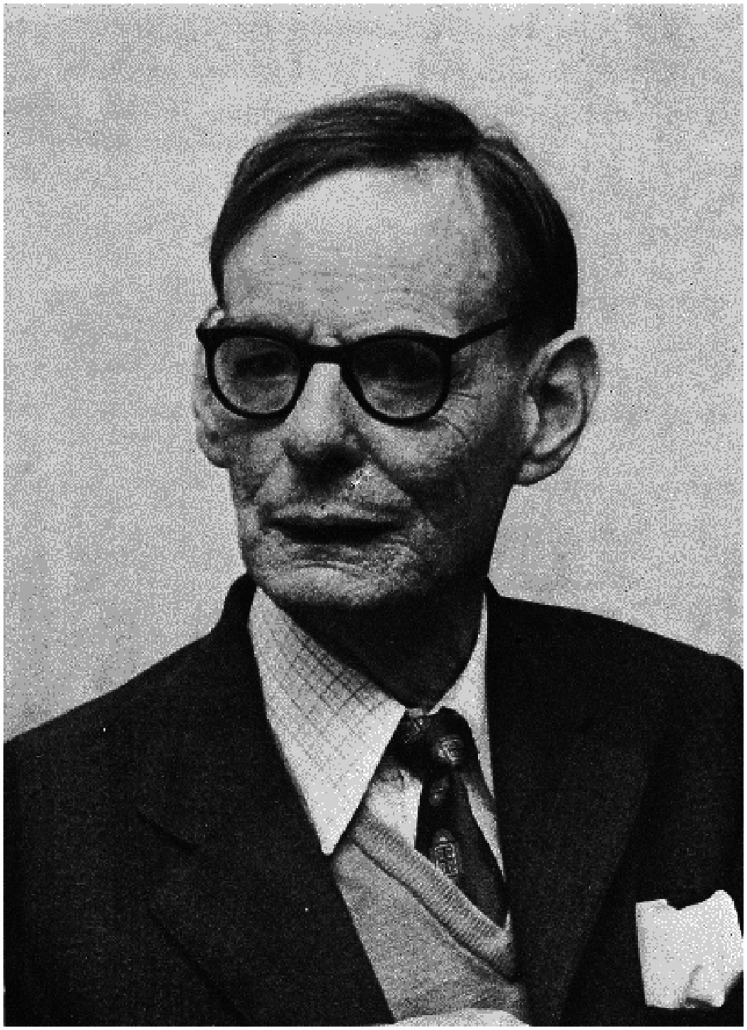
G. C. Grindley. Reproduced from Zangwill (1977).

In 1928, Grindley moved to Sir Frederic Charles Bartlett's Psychological Laboratory at Cambridge where he soon became a university demonstrator. In 1933, he became the third University Lecturer of the laboratory (Bartlett, 1937).^10^ At Cambridge, his interests diversified to topics in human vision such as the motion after effect (Grindley, 1930b; Grindley & Wilkinson, 1953) and various aspects of peripheral vision (Grindley, 1931a; Grindley & Townsend, 1968). Perhaps because of the complex apparatus needed for his work in physics (Cox & Grindley, 1927), Grindley was adept at building apparatus for experiments (Grindley, 1933, 1955) and classroom demonstrations (Grindley, 1930a, 1931b).^11^

Then came the impending threat of the Second World War, and with it a rapid expansion of the UK's RAF. In an effort to concentrate research in aviation medicine, the Air Ministry set up the RAF FPRC in 1939. This committee was a collaboration of the RAF Physiological Laboratory and the Cambridge Psychological Laboratory, among others, with the remit to study the medical needs and general wellbeing of aircrew, factors that influence operational efficiency, as well as the methods of personnel selection and training. In a discussion in 1939^12^ between the Chief Executive Officer of the FPRC Air Commodore H.E. Whittingham and Professor F.C. Bartlett (recorded in Whittingham, 1939), Grindley was proposed^13^ as the most suitable person to conduct a study of the current methods of training and instruction of flying personnel due to his “specializat[ion] in problems of training in physical and mental skill” (p. 2).

Grindley thus set out to understand how experienced pilots fly an airplane, with a view of being able to select the most suitable pilot cadets and to efficiently train the new recruits. One important aspect he focused on is the landing of an airplane, a difficult phase in flight, and specifically how pilots make the judgment of height above the ground, presumably to correctly time their landing flare. As discussed above in the “J.T. MacCurdy” section, Grindley first mentioned (recorded in Proposed psychological research, 1940) and briefly discussed (Grindley, 1940) MacCurdy's theory of aircraft landing in 1940 and stated that he hoped to later submit a report detailing the theory, its geometrical properties and its relevance to the RAF. This report was submitted in 1942 as FPRC report 426, after Grindley had finished reports on the other parts^14^ of his remit with the FPRC. In it, Grindley (1942) lists five cues that might afford the pilot to judge his height above the ground. These are convergence and retinal disparity, apparent (retinal) size of objects, perspective cues, clearness of detail and the retinal motion of the world, what Grindley calls the “apparent movement of objects in the visual field.” While he quickly rejects some of these cues as likely ineffective during the landing of aircraft, he goes into detail when discussing the apparent movement of objects in the visual field. It is perhaps best to let Grindley (1942) introduce this cue himself (underline his):

*The way in which such apparent movements may afford clues for the judgment of height is roughly as follows. If the aeroplane is descending at a constant speed, constant angle of descent, and constant attitude (as in a normal glide on a still day), the point on the ground towards which the aeroplane is flying will be stationary in the pilot's field of view. (i.e. it will be what is called a “zero point”). All other points will appear to be spreading out from this point, at various (retinal) velocities. These velocities will increase as the aeroplane descends … in such a way that they maintain a constant pattern; and the apparent velocity at any one point in the visual field … will be inversely proportional to the aeroplane's height. Thus, if a pilot can estimate this velocity to within 10 per cent, he can estimate his height to within 10 per cent under these conditions. … [Furthermore, the pilot should] be able to infer his angle of descent from the position of the zero point, and the general pattern of velocities. (p. 2)*

**Figure 3.**
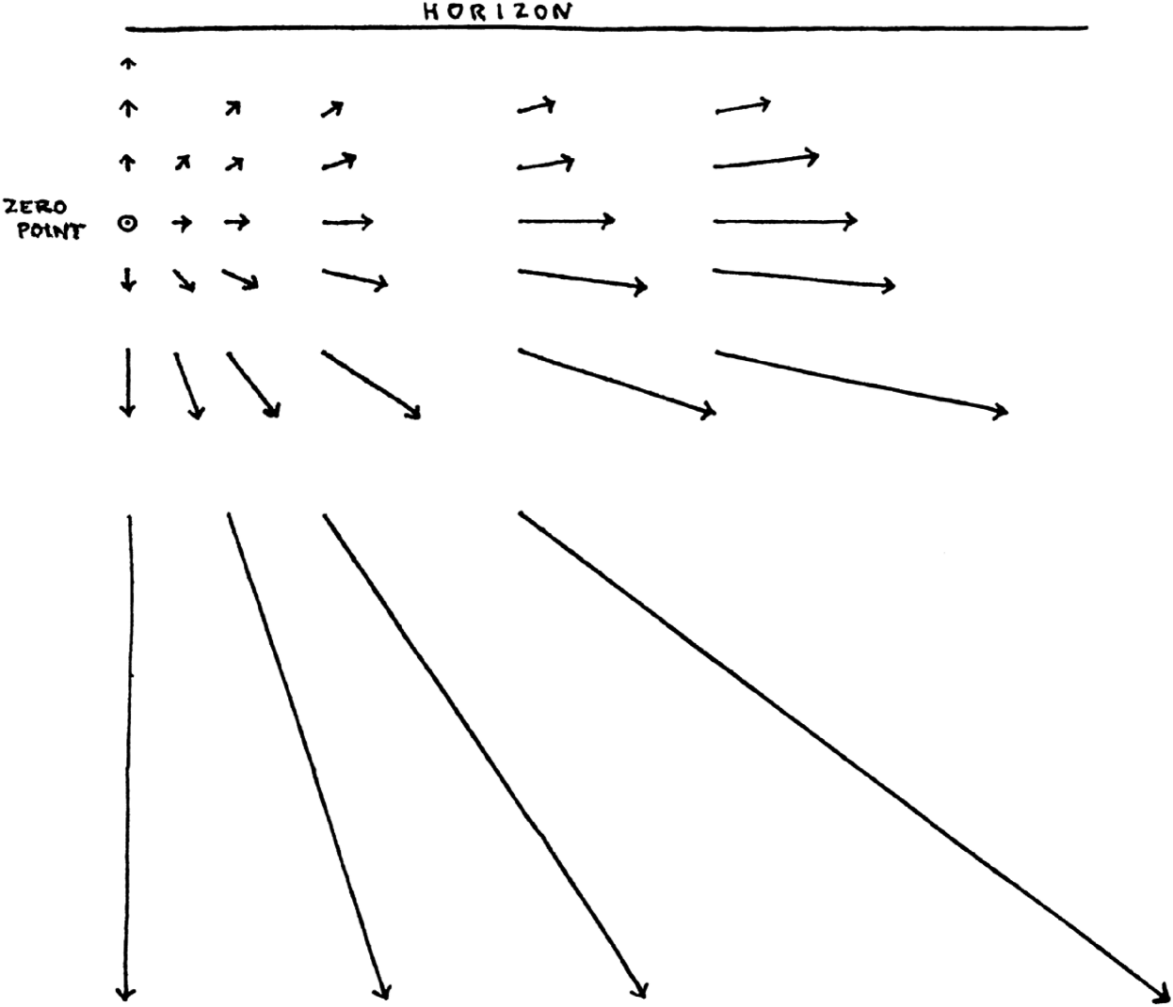
Hand-drawn figure depicting the visual velocities of points in the world during descent at an angle of 1 in 5 (11.3°). The arrows represent the direction and relative magnitude of optical velocities of points on the ground corresponding to the tails of the arrows. Reproduced from Grindley (1942).

Figure 3 shows an example pattern of velocities as drawn by Grindley. It is important to note that Grindley (1942) proposes that the velocity of single points is a cue for altitude. He does not appear to accord additional value to the pattern of velocities.

Grindley (1942) goes on to clarify that he does not suggest that a pilot makes conscious and deliberate estimates of visual velocity. Instead, Grindley takes the visual velocity of a point as a direct cue that affords judgment of height in a similarly direct way as the other cues listed previously. In fact, he notes that making judgments of height from retinal velocities has the advantage over the other cues “that the velocities in any part of the visual field will be independent of the nature, size, or clearness of the objects on the ground” (p. 2).

In an appendix to his report, Grindley (1942) develops mathematical expressions for the retinal angular velocity of world-fixed points due to translational self-motion and due to rotations about an axis. In this appendix, he demonstrates awareness that during translation, the retinal velocity of a world-fixed point depends on both the speed of translation, and the angular separation between the world-fixed point and the axis of translation. He furthermore notes that when the observer undergoes both translation and rotation, the retinal velocity of the point is the sum of a component due to the translation and a component due to the rotation. He notes that while the translational component depends on distance, the rotational component does not. The implication of this is that the rotational component in the retinal velocity of the point is constant irrespective of the distance of the point (c.f., Longuet-Higgins & Prazdny, 1980), although Grindley did not explicitly state this. In his discussion, Grindley (1942) emphasizes that these formulae for the translational and rotational components of retinal motion describe the sensory stimuli received by the pilot, but not what is perceived by the pilot. Grindley states that the conscious perception of the motion of points on the ground will be different, due to phenomena such as phenomenal regression (Thouless, 1931a, 1931b, 1933) and constancy.

Since in his theory the visual velocity of points in the world affords the pilot to judge his height above the ground during a landing, Grindley’s (1942) next step was to perform a series of experiments, to test whether observers could estimate the speed of objects with sufficient accuracy to support this judgment. Grindley had observers view a kymograph, whose rotating drum was wrapped with a sheet of green paper covered with irregularly shaped ink blobs, through a tube such that the field of view was about six degrees. In one experiment, before the start of a block, subjects were shown one of five standard velocities (0.4, 1.5, 4.5, 13.5, or 40.5 °/s) and were asked to remember this velocity. Then, for ten trials at each standard velocity, the experimenter accelerated the drum from zero in an irregular pattern and the observer was asked to say “stop” when he thought the standard speed was reached. Feedback was given each trial. It was found that the observers reproduced the standard more accurately for the highest velocity than for the other standard velocities. In other experiments in which a single display containing a range of velocities was used (the viewing tube was aimed off the axis of the kymograph drum), it was found that the ability to estimate the velocity of the field improved rapidly with practice with feedback. With 10 trials per day, observers attained average errors of 5% on the third day. Lastly, Grindley reported that almost all observers initially showed the tendency to overestimate the velocity of the field and call “stop” too early.^15^

His finding that human observers can estimate visual velocities with an error of 5% after only about half an hour of training led Grindley to conclude that the pilot would be able to estimate his height from the retinal velocities. As Grindley (1942) concludes (underline his):

*But if it is assumed that the degree of accuracy attained in these experiments can also be achieved in the air, the experiments would suggest that a pilot could land an aeroplane safely on the basis of velocity clues alone. … The fact that rather high velocities were estimated more accurately than low velocities suggests that parts of the visual field some distance from the “zero point” may be of special importance in the accurate estimation of height. (p. 3, emphasis his)*

Lastly, to return to his remit with the FPRC, Grindley (1942) discussed the implications of his theoretical and empirical findings for the selection and training of pilots. Regarding his experimental results, he notes that it should be possible to develop a selection test probing the ability of the subject to estimate visual velocities. He, however, notes that due to restrictions on test duration, it would be desirable to devise a test that assesses depth estimation from visual velocities and the other monocular cues in combination. Grindley states that this could be achieved with an inclined platform mounted on a moving trolley that approaches the observer. He also notes that such a device could be used for training if it allows for simulating various angles of descent. Lastly, Grindley (1942) observes that “cinematograph films taken from the position of the pilot's eye during good and (intentionally) bad landings [could be of instructional value]. Repeated viewing of such films, with suitable commentaries, might be expected to save a certain amount of time in dual flying, both in the case of elementary flying training and in changing over to new types of aircraft” (p. 4).

It is of interest to note that while Grindley's report shows that he was well-aware that judging the distance of points on the ground, and by extension aircraft altitude, requires knowledge of airspeed and the angle of separation between the direction of flight and the ground points, he did not discuss how these quantities might be available to the pilot making the judgment. While the angular separation is perceptually available when using the zero point as a reference for the direction of flight (the quotes above show Grindley was well-aware of this), airspeed would only be available from an instrument in the cockpit. As such, the perception of absolute height above the ground from the optical velocities could not be a rapid perceptual estimation, but must involve conscious calculation. I am enticed to ask why Grindley did not dwell on this difficulty but instead spent considerable effort researching how well observers can judge visual velocity per se. From the content of his report, I think it likely that Grindley viewed the problem of landing an aircraft as *recognizing* the correct height above the ground to flare, not as a problem involving absolute height perception. Indeed, Grindley's associationist lens shines through when he writes that “[The perfect aeroplane landing] will obviously depend … on the ability of the pilot to judge (or rather re-act correctly to) his height above the ground” (p. 1, emphasis mine). “[The pilot] has been shown the correct height for making any reaction … and then … he has to recognize the moment when he reaches this height again” (p. 1, emphasis mine)., and “What is suggested is that visual velocities may in fact be important clues determining his reactions” (p. 2). These quotes suggest that rather than positing that pilots perceive absolute height from the optical motions of the world, Grindley took the view that the optical motions of points in the world are likely informative for the pilot to determine his actions during landing. To support this point, Grindley showed that (1) during a descent at constant speed and glide angle there is a reliable association between the visual velocity of a point and the aircraft's height above the ground, and (2) this information can be picked up since pilots are able to judge (and thus recognize) different visual velocities quite accurately.

Grindley’s (1942) report remains as the only written work he has produced regarding the visual basis of the landing of aircraft, or any other aspect of self-motion perception. It is unknown why he did not pursue this topic further.

## J.J. Gibson

James Jerome Gibson (*1904–†1979, Figure 4) was a man who early in his career was influenced by some of the greats of his time, only to have a great and lasting influence himself. Never one to shy away from radical proposals, Gibson's seminal theories have shaped research in visual perception and beyond, even though some remain as controversial today as when they were first formulated. There are many aspects to the man and his work, but fitting with the theme of this article, I will mainly focus on his development of optic flow as information about self-motion and for guidance of locomotion. First, however, I will give a brief overview of his academic and intellectual life leading up to his work on optic flow, drawing mainly from the various biographical works that are available (Gibson, 1967, 2002; Hochberg, 1994; Lombardo, 1987; Reed, 1988).

**Figure 4.**
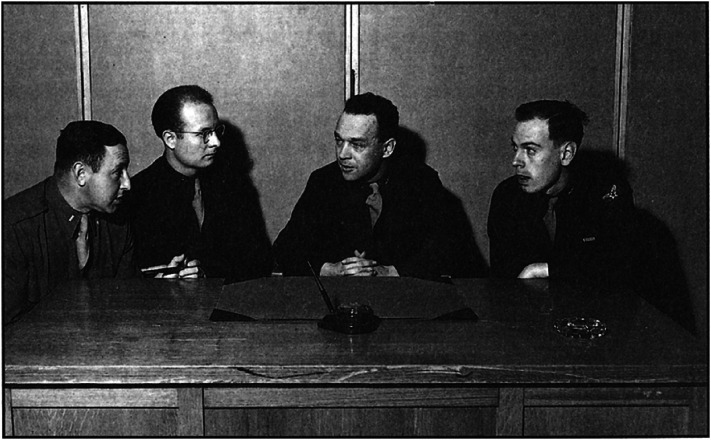
Members of the psychological test film unit. From left to right, Lt. Ralph Eisenberg, Capt. George Lehner, Capt. James Gibson, and Lt. Robert Gagné. Reproduced from Gibson (2002).

Gibson was somewhat of a loner until he found a place where he felt at home when he took up acting near the end of high school. Until his progressive deafness made it impossible some 20 years later, Gibson would seek out roles in the intimate theatre: “my proudest moments have been obtained on the stage. The achieving of a dramatic role, the expressing of a character, has given me deeper satisfaction than the playing of any of the other roles that an academic career affords—or the military, scientific, professional, or administrative roles” (Gibson, 1967, p. 127).

After high school, Gibson enrolled in Northwestern, and transferred to Princeton after his freshman year. Although he would major in Philosophy, he developed an interest in psychology in his senior year when he took a course in experimental psychology under Herbert Sidney Langfeld, who had worked in Germany with Wilhelm Maximilian Wundt and Carl Stumpf and had just been hired from Harvard in 1924. Langfeld offered Gibson and two other students from the class, Harold Schlosberg and Charles Bray, assistantships for graduate study in psychology. The Princeton psychology department would soon be reinforced by Edwin Bissell Holt, whose motor theory of consciousness and cognition would leave a great impression on Gibson: “It was a more elegant theory than that of any other behaviorist. For thirty years I was reluctant to abandon it” (Gibson, 1967, p. 129).

Gibson's dissertation (1929), completed in 1928, was a rebuttal of a study of visual memory of outline forms by Kurt Koffka's student Wulf (1922), giving a behaviorist instead of a Gestaltist account of his experimental results. Soon after completing his dissertation, Langfeld had arranged a job for Gibson as a lecturer at Smith College, starting in the fall semester of 1928. Koffka himself had just arrived there from Germany to take up a research professor position and he promptly opened a weekly seminar that Gibson would attend regularly until 1941. It was thus that Gibson became intimate with Koffka's ideas and absorbed his encyclopedic knowledge of perceptual psychology. As Gibson later stated (1967, p. 131), “Koffka, along with Holt, was a main influence on my psychological thinking.”

At Smith, Gibson's self-critical attitude and his drive to appraise the dominant psychological theory quickly became apparent in his first independent studies. By showing that a curved line looks less curved over time when one simply stares at it, he challenged the then prevalent sensory empiricist doctrine, which held that for each specific stimulus there is always the same percept and that this percept is learned through correlation of the sensory input with tactual experience and other motor interactions with the physical world (see Reed, 1988 for a discussion). Gibson's experiment (1933) not only showed that there is no fixed one-to-one mapping between sensation and percept, but also that visual percepts can change without any intervening behavior—a shocking discovery for an empiricist and behaviorist.

Gibson soon ran into further shortcomings of contemporary theories of visual perception when he and his friend Lawrence Edgar Crooks, an automobile engineer, tried to apply them to automobile driving in 1937. These theories could not describe how one visually steers a car, for this behavior could not be described in terms of atomistic psychophysics. Taking Lewin's (1936) topological psychology for a spin, Gibson and Crooks (1938) developed a dynamic theory of driving which emphasizes an egocentric account of how the path of safe travel is perceived as a function of passageways, such as roads, and barriers, such as obstacles. Driving was then the task of remaining in the center of this path of safe travel while appropriately adjusting speed. Although the “paper was not spectacular” (Gibson, 1967, p. 134),^16^ with the entry of the United States in the Second World War, Gibson would soon be working on similar problems again when he was asked by the U.S. Army's Air Forces to examine the control of aircraft.

### Gradients and Distance Perception

Before the United States was drawn into WWII, American psychologists were already organizing themselves for wartime service. In 1940, Gibson first joined the Subcommittee for Perceptual Problems of the Emergency Committee in Psychology of the National Research Council, for which he produced a literature survey concerning the “organization of the visual field with special reference to camouflage” (a part of Fernberger et al., 1941). Soon after, in 1941, Gibson joined the Army, as did many other psychologists during the war (Marquis, 1944). He left Smith College for Washington DC where the Army Air Forces were organizing the Aviation Psychology Program (see “Aviation Psychology Program,” 1943; “Present organization of Aviation Psychology Program,” 1945; and especially Flanagan, 1948 for overviews of this program). As a part of this program, Captain (later Major and eventually Lieutenant Colonel) Gibson would work on the selection and training of aircraft crew. Gibson was first made chief of the Perceptual Research Unit, which was set up in July 1942 at the Army Air Force Flying Training Command headquarters in Fort Worth, Texas. This group administered both printed and motion picture group tests. To continue work on motion pictures as a testing and training medium, starting in October 1943 Gibson then spent a further two and one-half years at the Santa Ana Army Air Base in California as head of the Psychological Test Film Unit (see “History, organization, and research activities, Psychological Test Film Unit,” 1944^17^; and Gibson, 1947 for overviews of the unit and its work).

From an inventory of the causes for eliminating cadets from flying training (Flanagan, 1942; see also Gibson, 1947, chap. 9), it became clear that important visual skills for flying include the ability to perceive and maintain spatial orientation, as well as the ability to estimate ego-speed and the distance of objects in the world. However, the nature of space and self-motion perception had to be understood first in order to develop effective selection and training methods with which these abilities can be selectively gauged and improved. As Gibson had already established during his earlier work (Gibson & Crooks, 1938) that contemporary perceptual theories had little to say about these perceptual abilities, he developed his own theory of how spatial orientation and ego-motion, as well as the distances of objects are perceived. The binocular cues to depth, which at that time were thought to be primary in perceiving depth, do not operate at the scales of distance experienced when operating aircraft (Gibson, 1947). As they thus cannot underlie the experience of the third dimension by pilots, a new basis for the perception of distance was needed.

Gibson responded to this challenge by formulating his ground theory of space perception (see Sedgwick, 2021, for an extensive discussion), which he first described in a chapter that he wrote in collaboration with N.M. Glaser, a member of the Psychological Test Film Unit, and included in the extensive report he produced after the war on the work of his unit (Gibson, 1947, chap. 9). His theory emphasized stimulus information that is directly available in the retinal image, and which specifies a ground plane that meets the sky in the horizon: “the problem of three-dimensional vision, or distance perception, is basically a problem of the perception of a continuous surface which is seen to extend away from the observer. All spaces in which we can live include at least one surface, the ground or terrain. If there were no surface, there would be no visual world, strictly speaking” (Gibson, 1947, p. 185).^18^

Specifically, Gibson posited that the ground surface provides a “sensory continuum of distance, *as such*, which, once visible, determines how distant all the objects within it are” (Gibson, 1947, p. 186, italics his). Gibson recognized that a continuous surface receding in depth is specified by the continuous differential stimulation present in the retinal image, which he referred to as a gradient. In his report, Gibson listed several of such retinal gradients, each of which provides a continuous retinal correlate of distance: the gradient of texture, the gradient of size of similar objects, aerial perspective due to the transmission of light through the atmosphere, the gradient of binocular disparity, and the gradient of retinal velocity of the ground during self-motion, which he called motion perspective (Gibson, 1947, pp. 188–193).

### The Motion Perspective Gradient

The gradient of motion perspective is special in that it not only provides information about distance, but also about self-motion. Gibson would recall in his autobiography that perhaps the earliest time he was aware of such visual motion patterns was when he was eight years old and frequently travelled on trains because his father was a surveyor for the railroads. From the train, he would notice how the world “seemed to flow inward when seen from the rear platform and expand outward when seen from the locomotive” (Gibson, 1967, p. 127). However, the story of Gibson's elaboration of motion perspective starts during his time with the Army Air Forces.

**Figure 5.**
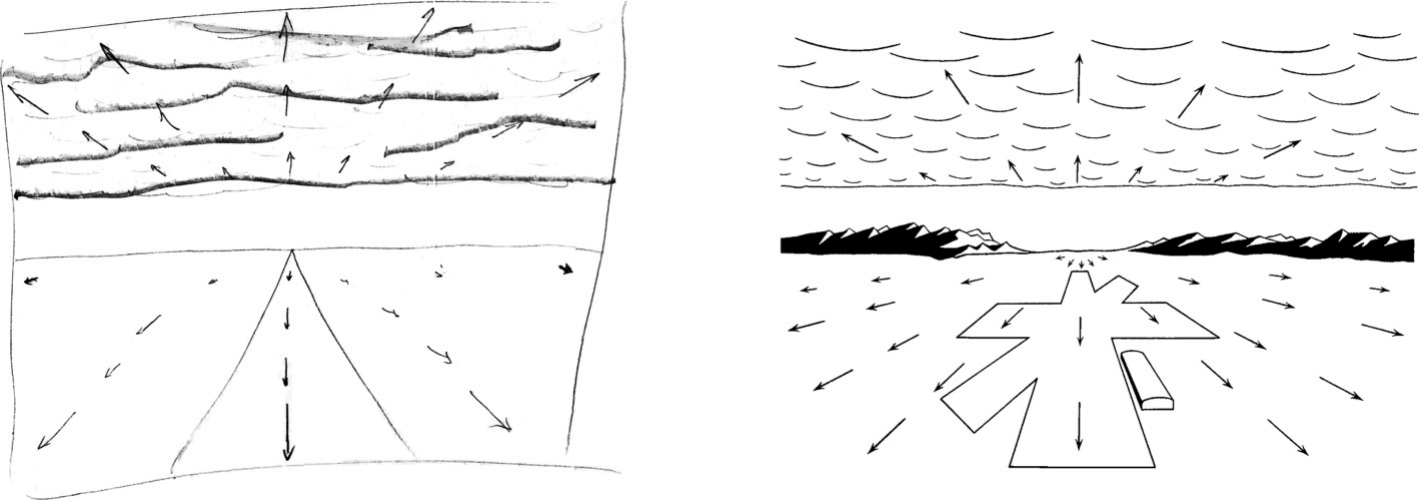
Left: draft, and right: final version of a figure depicting the motion perspective experienced during level flight while the sky is overcast. The left figure was drawn in 1942 and is reproduced from Gibson’s notes (Gibson, 1942–1945). The right figure is reproduced from Gibson (1947).

**Figure 6.**
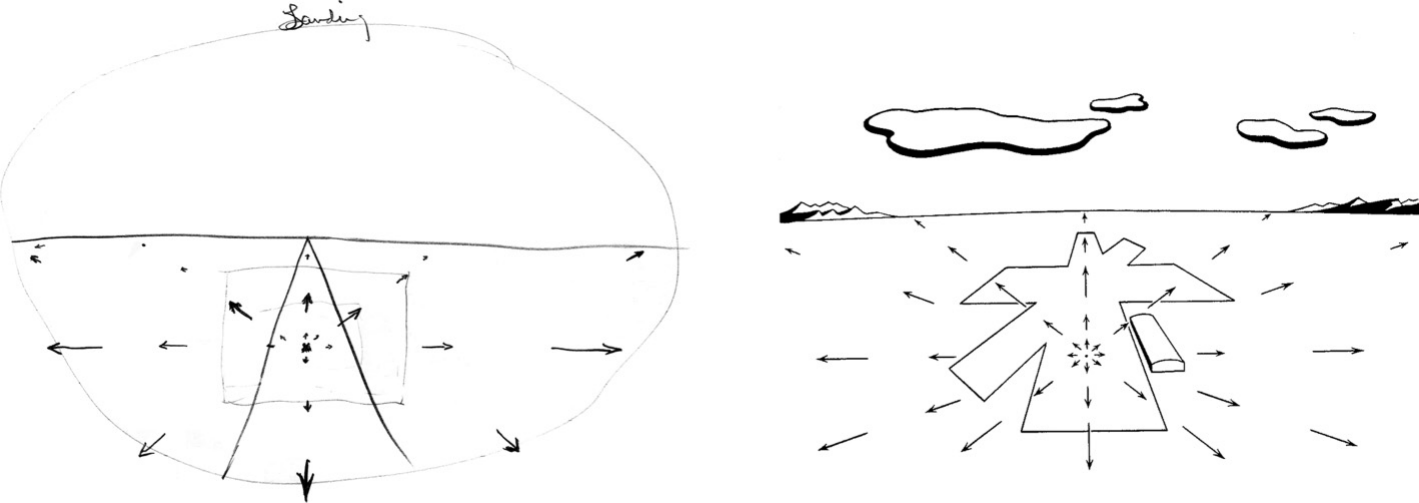
Left: draft, and right: final version of a figure depicting the retinal motion gradients experienced during a landing glide. The left figure was drawn in 1942 and is reproduced from Gibson's notes (Gibson, 1942–1945). The right figure is reproduced from Gibson (1947).

Notes that Gibson wrote on the topic in 1942 (Gibson, 1942–1945) provide us with the earliest source of his thinking on optic flow. They consist of a summary of Grindley’s (1942) mimeographed report, which Gibson submitted to his commanding officer on August 12 as abstract series no. 27, with the title “*The Perception of Visual Velocity*.” In this summary, Gibson used Grindley's terminology (see the “G.C. Grindley” section above). He furthermore made two comments on Grindley's report. First, although the principles of Grindley's analysis of the retinal motion are valid for any looking direction, Grindley only discussed the forward view during landing. Gibson noted in his abstract that the inverse relation between retinal velocity and distance of points on the ground holds for any looking direction. In the second more extensive comment, Gibson found the large increase in accuracy with practice and feedback that Grindley observed in his visual velocity estimation experiments is “very suggestive for perceptual research.” However, he thought that these effects are likely due to the “overcoming of a strong tendency to a constant [underestimation] error, which may have been partly induced [by the method, as] the standard velocity was always approached from lower velocities.” Separate from his summary of Grindley's report, Gibson's notes also contain drafts of most of his well-known figures illustrating the motion perspective gradient experienced when looking out the window of an airplane, which constitute perhaps the earliest preserved illustrations of optic flow by Gibson (see Figures 5 and 6).

A section in chapter 9 of Gibson (1947) contains the first publication of Gibson's theories of the visual perception of self-motion in the real world. Expanding further on the motion perspective gradient, Gibson notes that as motion not only has a magnitude but also a direction, the retinal motion pattern not only provides information about the distance of objects in the world, but also about the direction of locomotion of the observer. “In addition to the fact that the velocity of this retinal flow approaches zero for very distant objects and vanishes, therefore, at the horizon, it also approaches zero and vanishes at two specific points in the visual world—the point toward which the observer is moving and its opposite, the point he is moving away from”^19^ (Gibson, 1947, p. 221). During locomotion parallel to the ground plane, the point toward which the observer moves is on the horizon. Gibson continues by noting that “optically speaking … the world expands radially outward as the observer moves into it, and, assuming he looks backward, contracts radially inward as he moves away from it” (p. 222). Gibson recognized that this expansion, created by the fact that “the direction of all retinal velocities is radially outward from the point toward which one is moving” (p. 222), is a cue with which the direction of self-motion can be perceived. He called it the “center of expansion” cue.

Next, Gibson discusses the effect of eye movements on the retinal motion during self-motion. He notes that eye movements displace the retinal image, but do not cause it to undergo deformation. In other words, an eye movement, such as smooth pursuit of a point on the ground, adds a constant velocity to the retinal motion of all points in the visual world. The addition of such a constant does not alter the motion perspective gradient, as the differential velocity between points in the world remains unchanged. Gibson thus concluded that, as the observer has the same visual experience of locomotion through space instead of deformation of a flat image regardless of whether he fixates or moves his eye, “the effective stimulus for such perception is the gradient of velocities in the retinal field—the direction and rate of change of velocities along a retinal axis—rather than the velocities themselves” (Gibson, 1947, p. 225).^20^ It becomes apparent from a note Gibson penciled underneath one of the draft figures from 1942 (Gibson, 1942–1945) that he realized very early that only the retinal stimulus but not the percept is affected by eye rotation: “fixation (or pursuit) has basically nothing to do with the perception of movement. It does have everything to do with the retinal stimulus for movement” (underline his).

Last, Gibson discusses the application of his theory of motion perspective to the problem of landing an airplane. When landing an airplane, the observer approaches the ground plane, making his ability to judge distance and direction of self-motion all the more important. During landing, the center of expansion is now on the ground. Although the retinal motion pattern remains symmetrical, the gradients are quite different from those experienced during locomotion parallel to the ground plane as the retinal motion now vanishes both at the horizon and at the center of expansion (see Figure 6). The center of expansion indicates the current aim point of the landing glide, while the angular distance between the center of expansion and the horizon corresponds to the angle of glide. Furthermore, Gibson noted that altitude and groundspeed are specified by the overall rate of the expansion pattern, although “the visual cue for speed and one of the cues for altitude are, if this analysis is correct, the same stimulus” (Gibson, 1947, p. 229), making judgment of either in isolation difficult. Lastly, Gibson states that “the theory [of motion perspective] needs to be formulated more exactly by the use of methods similar to those of projective geometry. Only a beginning has been made in such a task” (p. 229). He would finally do so in Gibson et al. (1955), which is discussed in the “Further Elaboration during the 1950s” section below.

### Visual Perception of Self-Motion Direction: The First Experiment

Although many details of Gibson's theory remained to be worked out, the new understanding of the importance of motion perspective in the landing of airplanes allowed Gibson to create the Landing Judgment Test (the development of this test, CP505E, is described in detail in Gibson, 1947, pp. 70–72, 230–240), to be administered to prospective cadets together with a battery of other tests. A motion picture test of distance to the landing point employing a temporal judgment was also planned, but never completed (see Gibson, 1947, p. 198). As this landing judgment test constitutes to my knowledge the first task designed to test the ability to visually perceive self-motion direction, a discussion in some detail is warranted.

**Figure 7.**
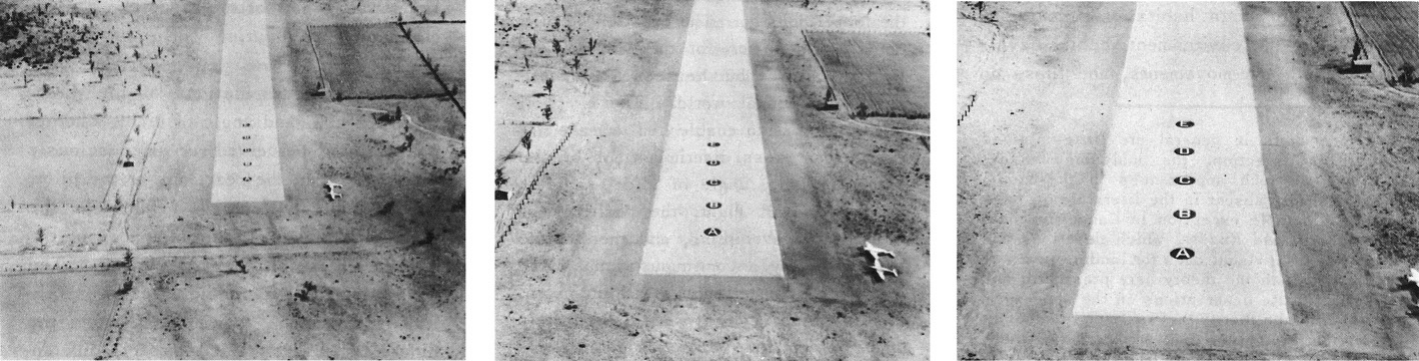
Three frames from the landing judgment test film, showing the view of the runway from 1000 feet, 500 feet, and 250 feet, respectively. The depicted approach is toward point D. Due to issues with framing of the original images, I have arranged the panels such that the position of point D is aligned across the images. Reproduced from Gibson (1947).

The Landing Judgment Test consists of a film that depicts the view from an aircraft that descends at a constant angle of 30° toward one of five aim points on a grassy field at simulated speeds of 26, 35, or 52 miles per hour, yielding 15 clips of between 20 and 40 s in duration. The approach slope was an order of magnitude steeper than real world approach slopes and the approach was slower than obtained with real aircraft, but “this was believed to be essential for the purpose of obtaining adequate degrees of difficulty and variability in test items” (Gibson, 1947, p. 71). To eliminate the yawing and pitching motions experienced in a real airplane, which were found to be disturbing when viewed on film, and to enable the steep approach slope, the film was shot using a model airfield and a camera that moved along a track. The camera's field of view was 35° H × 26° V, but as participants were seated in groups in a room during the experiments, the size of their field of view covered by the motion picture varied. Assuming the Psychological Test Film Unit followed its own recommendations, the horizontal size of the display would have been between 29° and 10° (see Gibson, 1947, p. 50). The letters A to E were painted on the first third of the runway to indicate the five possible aim points (see Figure 7), and after viewing each clip, cadets were asked to select the correct aim point out of these possibilities.

The final test was administered to 1,200 cadets at Keesler Field, but the results were not yet available at the time when Gibson was writing his report in 1945 to 1946. Gibson (1947, pp. 232‒238), however, does present data obtained from pilot tests with groups of preflight students. All the pilot tests used the final motion pictures recorded as described above. In the recorded films, the camera was always aligned with the direction of travel, and as such the correct item always appeared in the center of the screen during the entire movie. A first test using the 15 clips in random order was therefore conducted to determine if this center confound could be avoided by offsetting the picture randomly upward or downward in relation to the frame of the movie. When this offsetting was performed, a group of 66 participants attained on average only 39% correct responses, compared with an average score of 63% correct obtained by a different group of 45 participants when the clips were centered within the frame of the movie.

A second test using offset pictures was conducted to examine the effect of explicit instructions and training on performance. The training and instructions told participants to judge the expansion of the scene, and to select the point at the center of the expansion, which is the point that does not move on the screen. Such training may seem odd for a selection test which would presumably endeavor to examine students’ innate aptitude in perceiving the position of the aim point. However, Gibson (1947) notes that it was presumed that new students would not already be familiar with the appearance of the ground during approach. The test therefore set out not to measure a student's original ability in judging the aim point, but “the aptness of the student in learning to make the judgments described after instruction and practice” (Gibson, 1947, p. 239). To examine the effect of the instruction and practice, the test was administered to two groups of preflight students. The first group of 66 participants were simply shown 45 trials and attained on average 39% correct responses, with no increase in performance from the first 15 to the last 15 trials. The second group of 84 participants first completed 15 trials of the test, and then received training for 15 trials during which they were given the instructions concerning the expansion cue and were told the correct answer after each trial. For a further 15 trials collected after the training procedure, it was found that the average percentage of correct answers rose to 48%, up from the 37% correct responses that this group attained in the first 15 trials.

Then, the final test was constructed, for which the 15 video clips were turned into 60 items. For this test, two different cuts were made from each of the video clips to create easier and more difficult trials. These cuts were made such that 10-s clips were created that ended at two distances from the runway to manipulate the rate of expansion in the video (for the effect of translation speed on perception and control of self-motion direction, see Chen et al., 2018; Warren et al., 1988). The easier trials, which contained higher flow rates by taking the last 10 s from each video clip, constituted the first 30 trials of the test and the remaining 30 harder trials constituted cuts from earlier in the clips. In testing on an unknown number of participants, it was found that the easier items yielded scores between 55% and 70% correct while the harder items yielded scores of 45% to 55% correct.

While the accuracy of participants in identifying their aim point from Gibson's film might seem low (even if above the chance level of 20%), it must be interpreted noting that the test was specifically designed to yield this medium level of performance so that there was sufficient range to screen out outstanding as well as bad performers. It furthermore needs to be considered that the projected image of the film was small, and research has since shown that self-motion perception benefits from large field of view displays (see, e.g., Koenderink & van Doorn, 1987 for a mathematical treatment and Li et al., 2009 for experimental work). Thus, the results of Gibson's experiments indicate that observers can make reasonably adequate estimates of self-motion direction from optic flow.

### Further Elaboration During the 1950s

In the remainder of this discussion, I will focus on the key developments in Gibson's thinking about optic flow in the 1950s. Between completing his work for the Army Air Forces and the publication of his first major book, “*The Perception of the Visual World*” (Gibson, 1950), Gibson mentioned motion perspective in one other publication, a book chapter published in 1948. When it comes to motion perspective, in this chapter Gibson (1948) did not deviate from the optical facts he presented in 1947, nor did he change his terminology (though he did introduce a new gradient, which would now be called shape from shading, Gibson, 1948, p. 178).

This chapter however contains the beginnings of an effort that would come to full fruition in Gibson's book, “*The Perception of the Visual World*” (Gibson, 1950). In this book, Gibson develops a new theory of perception out of the perceptual facts he described in Gibson (1947). Building on his ground theory, Gibson detailed a theory of perception of the active observer. In daily life, the retinal image is in continuous flux as the observer explores the world through locomotion and eye movements. Gibson realized that the physical exploratory behavior and the change of the retinal image are reciprocal: locomotion generates flowing deformations of the retinal image and this flow in turn lawfully and precisely specifies the observer's locomotion along with the layout of the world. Gibson thus posited that flow provides for a visual kinesthesis in that it specifies the perception of the movement of the observer through space, as well as his position in space.

In this book, Gibson (1950) revised his terminology. He now used the term motion perspective in the sense of the total pattern of retinal motion, and started consistently using the term flow to describe the changes that the retinal image undergoes during self-motion, thereby coining a term that is in regular use today in both the cognitive science and computer vision communities (note that he is seen using the more specific term “optical flow” in Gibson et al., 1955). Gibson (1950) also introduced the term focus of expansion^21^ to refer to what he called the center of expansion in 1947. At this time, his theory of how flow indicates the direction of locomotion had evolved. He stated that it is not the focus of expansion itself, that one point in the world which remains stationary in the visual field that specifies the direction of self-motion. Instead, Gibson realized that the direction of self-motion is implicit everywhere in the flow pattern and that the observer can thus perceive his direction of self-motion even if the focus of expansion is not in view (see, e.g., Crowell and Banks, 1993; Warren, 1976; Warren and Kurtz, 1992).

This understanding of how the directional structure of the flow indicates self-motion direction enabled Gibson to formulate an hypothesis of how flow is used for guiding locomotion, such as steering a car: “it is less a matter of *aligning the car with the road* than it is a matter of *keeping the focus of expansion in the direction one must go*” (Gibson, 1950, p. 128, italics his). In 1950, this hypothesis was just a single sentence. During his stay in Oxford on a Fulbright fellowship in the academic year 1955–1956 (see Gibson, 2002), Gibson further developed this theory. In the resulting paper, Gibson (1958) clearly formulated what is now called the perception-action feedback loop (see Warren, 2006) when he said that the flow experienced during self-motion “is an invariable accompaniment of locomotor behavior and therefore provides ‘feedback’ stimulation for the control and guidance of locomotor behavior” and described formulae for how specific behaviors are visually controlled (tentative formulations of both appeared in Gibson, 1955). Gibson (1958) made several other key advances in this paper, such as complete descriptions of the optic array and what he later called affordances. Warren (1998) discusses these and other aspects of Gibson (1958) in detail as well as the body of work precipitated by it.

A further significant development came in 1955, when Gibson and his two more mathematically inclined colleagues Paul Olum and Frank Rosenblatt (1955) developed a mathematical analysis of the optic flow field.^22^ Up to this point, Gibson had only described flow phenomenologically and he now wished to properly analyze it from a geometrical standpoint to find out what optical information it contains about environment layout (relative distances), self-motion direction, and time to contact. To this end, Gibson et al. (1955) developed a detailed and general analysis of the angular velocities optically present at a point of observation that moves through a rigid world. Although they did not develop expressions for the direction of motion at each point (this always being away from the focus of expansion), they concluded that the optic flow pattern contains information about both the observer's position and motion as well as the surface's distance, slant, and shape. Note, however, that not all these conclusions were mathematically developed to demonstrate how this information is available from the optic flow (a detailed overview of later more complete and sophisticated analyses is given in Warren, 1982).

It is noteworthy that in this paper, Gibson et al. (1955) presented their results independent of eye position, instead giving complete hemispherical descriptions of the available optical motions. This is an important departure from his 1947 and 1950 books, where Gibson discussed patterns of retinal motion which carry the complication of additional motion components due to eye movements. Starting with the 1955 analysis of the optic flow pattern, by using these hemispherical descriptions Gibson adopted a representation in which he no longer considered retinal stimulation, but the pattern of light reflected to a stationary (or moving) point of observation—what he would later call the “optic array” (published in Gibson, 1957a; see also Gibson, 1957c, 1961; see Reed, 1988, chap 9 for a discussion of this development). This was the birth of Gibson's “ecological optics” (Gibson, 1957c).

A last development in Gibson's thinking during the 1950s that I will discuss is his adoption of continuous perspective transformations as the primary stimulus variable for object motion or self-motion perception (Gibson, 1957a), instead of the velocities associated with individual rays in the optic (or retinal) array. This is perhaps most clear in the following passage in Gibson (1957c) describing one of the “properties of optical structure” that may “carry information about the environment” (p. 5):

*Continuous transformations. A continuous series of perspective transformations of a closed contour and its component textural elements specifies a moving rigid object in the environment. A continuous non-perspective transformation of a closed contour and its elements specifies a non-rigid movement of a substance in the environment – a liquid surface, a vapor surface, or a viscous solid (which includes the animate movements of organisms). A continuous perspective transformation for the pattern of the whole optic array surrounding the station point of an observer specifies locomotion in the environment, that is, a change of station point [(* Gibson et al., 1955 *)]. (p. 7, underlines his)*

Gibson is seen here to consider the pattern of optical motions as primary and abandons any reference to individual motions within the patterns as stimuli for space or self-motion perception. In Gibson (1957a), it is furthermore noted that “a unifying hypothesis [for depth derived from optical motion and binocular stereoscopy] would be that the simultaneous disparity of binocular images is only a special geometrical case of the successive disparities of a continuous image,” since “both rest on the geometry of parallax, that is, the projection of a collection of objects in space to a point in space” (p. 292). In connection to this, Gibson noted that this led Tschermak-Seysenegg (1939), that is, the article I discussed in the “Tschermak-Seysenegg (1939)” section above, to group both sources of depth information under the term parallactoscopy (Parallaktoskopy). Lastly, it is interesting to note the parallels to the work of Dauser (1939) discussed above, who also saw perspective shifts as stimuli for both self-motion perception and the perception of a stable world.

## E.S. Calvert

The Irish engineer Edward Spence Calvert (*1902–†1991, Figure 8), described as “scrappy” by Gibson (letter of J.J. Gibson to K.M. Dallenbach, October 11, 1956; in Gibson, 1956), joined the Electrical Engineering Department of the Royal Aircraft Establishment in the United Kingdom in November 1928, working mainly on aircraft lighting (Calvert, 1948; “Edward Calvert,” 1991). In 1941, he became head of the illumination section, a post he would hold until he retired from the Royal Aircraft Establishment in 1967 (“Edward Calvert,” 1991). After having worked on topics such as cockpit lighting (e.g., Calvert, 1938, 1944, 1951), in 1944 (Calvert, 1947c) Calvert started working on the problem of developing high intensity approach and runway lighting to aid aircraft landing at the request of the Airfield Lighting Committee of the United Kingdom's Ministry of Civil Aviation. It is in this context that Calvert discovered the importance of retinal motion in airplane guidance, which led to the development of his parafoveal streamer theory.

**Figure 8.**
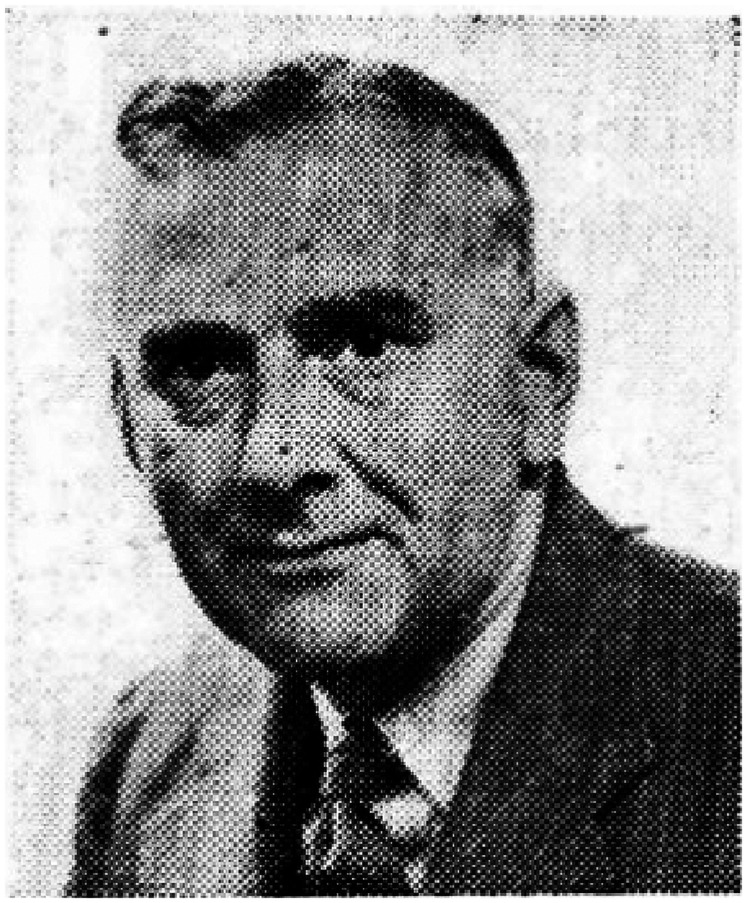
E. S. Calvert in 1949, taken when he received the Wakefield Gold medal from the Institute of Aeronautical Engineers for his work on airport lighting (see “Royal aeronautical society: Awards,” 1949). © Farnborough Air Sciences Trust, reprinted with permission.

The systems of approach and runway lighting in use in the early 1940s were sufficient to support all normal visibility operations, but none of these low intensity systems were designed for conditions of limited visibility (Calvert, 1947c). About 1944, radio aids became sufficiently developed to allow bringing aircraft down close to the runway regardless of visibility, opening the door to all-weather operation of airports. When landing in limited visibility, the pilot at first relies on his instruments and on radio aids such as ILS (Charnley, 1958, 2011) to bring him down to about 200 feet. Then, for the final part of the approach and landing maneuver, the pilot has to transition to visual guidance of the aircraft. Given that the pilot operates under great duress in low visibility conditions and is possibly fatigued, the risk of accidents is high if at this point the visual scene does not provide sensitive and unambiguous indications of aircraft state that are easily recognized without close examination or considerable practice. Therefore, new high intensity visual aids had to be developed to allow for safe completion of the landing maneuver in bad visibility.

**Figure 9.**
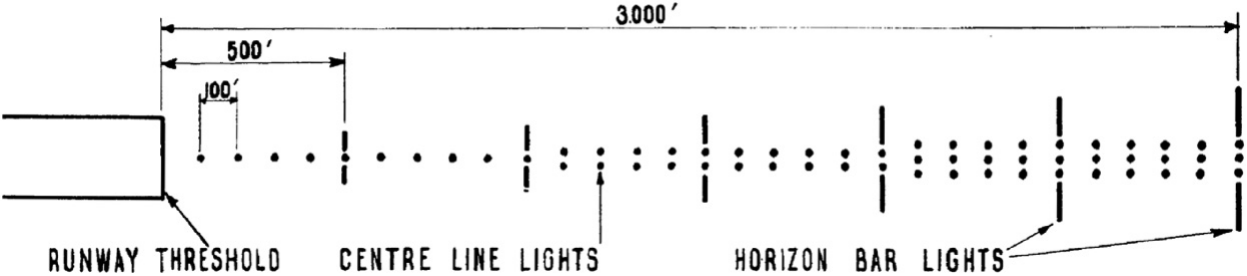
Plan of an example calvert centre line and crossbar system, as was installed in 1951 at Essendon, Victoria by the Australian Department of Civil Aviation. Distances are indicated in feet. © Civil Aviation Historical Society Inc., reprinted from Hart (1951), with permission.

Visual control of the aircraft's six degrees of freedom is a complex problem, and Calvert recognized that any approach lighting pattern must, by itself, be geometrically sufficient to provide unambiguous information about aircraft state. He argued that in order to allow for a systematic analysis of the problem and to enable a scientific discussion about possible solutions, a good description of the visual information provided by the landing aids is needed (Calvert, 1947c). For this purpose, he developed a method he called perspective analysis (Calvert, 1947a), which consists of constructing the pilot's view of the approach lighting pattern and the runway through a perspective projection procedure. He then applied this method to design an all-weather approach lighting pattern (Calvert, 1947b, 1947c, 1948), the Calvert Centre Line and Crossbar system depicted in Figure 9.

Calvert also used his analysis method to compare how airplane state is represented in the visual information provided by his and other proposed landing aids (Calvert, 1949, 1950). This analysis revealed that the visual information provided by most of the proposed new approach lighting patterns (see Calvert, 1949; Hart, 1951 for overviews) was subject to several ambiguities (e.g., confusion of the aircraft's angle of bank and lateral displacement^23^) and gave rise to several optical illusions that could easily lead to accidents (see e.g., Calvert, 1950, 1959; Cocquyt, 1953; Gibb et al., 2010; Pitts, 1967, 1969). Calvert's system suffered least from these kinds of problems. Thus, after several meetings (Draper, 1948; ICAO, 1949) of the International Civil Aviation Organization (ICAO) in which multiple approach lighting systems were discussed (see Hart, 1951; ICAO, 1948; Petzel, 1958 for overviews of the proposals under discussion at different stages), the ICAO adopted the Calvert system as an international standard in 1952 (Boussard, 1952). It is still in use on airfields today (Charnley, 2011).

From descriptions in these early analyses of the visual information available when landing an airplane, it is obvious that Calvert was aware of the visual motion patterns that occur during approach:

*If a certain small part of the pattern appears to increase in size while keeping the same shape and bearing, then the observer knows he is in motion towards that part of the pattern. (* Calvert, 1947a *, p. 2)*

*The only indication the pilot has of his aiming point is that objects nearer to him than [the aim point] where the approach path cuts the ground appear to him to move downward underneath the fuselage, while objects further away than [the aim point] appear to move upward towards the horizon. [The aim point] is, of course, fixed with respect to the horizon. (* Calvert, 1947c *, p. 9)*

*If an observer is in motion in a straight line towards a point in a pattern, that point will appear to him to be stationary in the pattern, and the points surrounding it will appear to move radially away from it. (* Calvert, 1949 *, p. 6)*

However, at that time, he held that the stationary point in the visual scene is used for visual guidance of the aircraft, not the motion pattern as a whole, even though he did observe that “the observer [can] find the point towards which he is moving with an accuracy which increases as he gets closer to it” (Calvert, 1949, p. 6) and thus did appear to appreciate that visual velocities play a role (see also Warren et al., 1988). The primacy of the stationary point itself in his thinking is however borne out by, for instance, his statement that “In good visibility, the pilot can judge his aiming point […] by noticing what point in the ground pattern remains at rest […]. In bad visibility, the pilot will be unable to see his aiming point, and will therefore have to use a different technique” (Calvert, 1949, p. 14) and his statement that very little vision around the stationary point, probably around 3°, is needed during approach (Calvert, 1949, pp. 6‒7).

As described in Calvert (1950), the realization of the significance of the visual motion patterns for the visual guidance of aircraft came in 1949 and developed from two sources of inspiration. First, in order to facilitate testing and discussion of the optical properties of the various visual landing aids under consideration, Calvert asked two members of his department to construct a flight simulator that could simulate low visibility approach and landing. The resulting mechanical simulator (Sparke & Ringe, 1949, 1950a, 1950b), known as “The Cyclorama,” provided the possibility to experience a continuous view of the lighting pattern from an aircraft cockpit during approach and landing. Demonstrations given on it helped convince pilots, engineers, and administrators of the effectiveness of Calvert's approach lighting system. Calvert (1950) recounts that the first clue to the importance of visual motion in aircraft guidance came in 1949 when examining the accuracy with which certain visual judgments could be made when performed on the Cyclorama. Calvert found that the accuracy of observers was higher than would be expected from merely noting the differences between successive views (he did not specify what he based this assertion on), which indicated to him that the observers appeared to obtain information from the kinematic qualities of the visual field.

**Figure 10.**
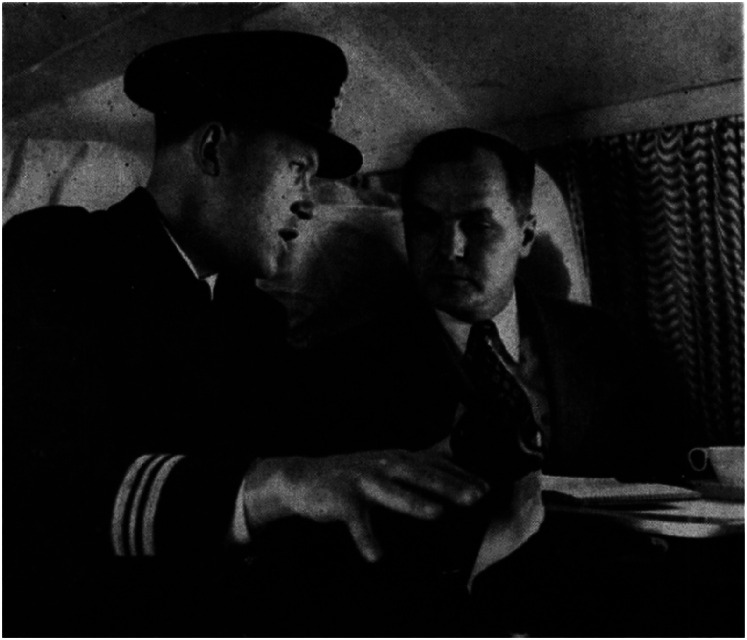
Captain Alastair Michael Adair Majendie. After service for the RAF during WWII, Majendie joined the British Overseas Airways Corporation (BOAC) in 1946 and rose to the position of flight captain for its de Havilland Comet I fleet in 1951 (“Mr A. M. A. Majendie”, 1970). With a de Havilland Comet I, he flew the first scheduled passenger jet service on May 2, 1952, from London to Johannesburg. Here Capt. Majendie (left) is shown during this maiden flight conversing with Mr. Cookman (right), author of an article about this flight (Cookman, 1952). For an account of BOAC's flight experience with the Comet I, see Majendie (1953). Reproduced from Cookman (1952).

Second, at the same time, Capt. Majendie (Figure 10) informed Calvert that he was convinced by personal experience that below heights of 150 ft., the pilot derives his most important impressions from the motion in the visual field. Calvert (1950) mentioned that Majendie showed him several films of pilots’ faces when they were making a landing. From these it was observed that when flying at low heights, the pilot often stares fixedly ahead for as long as a few seconds at a time (see also Calvert, 1954; Majendie in the discussion following Calvert, 1950; and Majendie, 1960). This fixation behavior was taken as evidence for the idea that visual motion patterns play a role in judgments of height and self-motion direction as staring fixedly ahead was believed to make it easier for the pilot to make these visual judgments. Calvert (1955a) clarified that the reason that pilots stare straight ahead in this manner is that only then does the streamer pattern look as I will discuss next.

**Figure 11.**
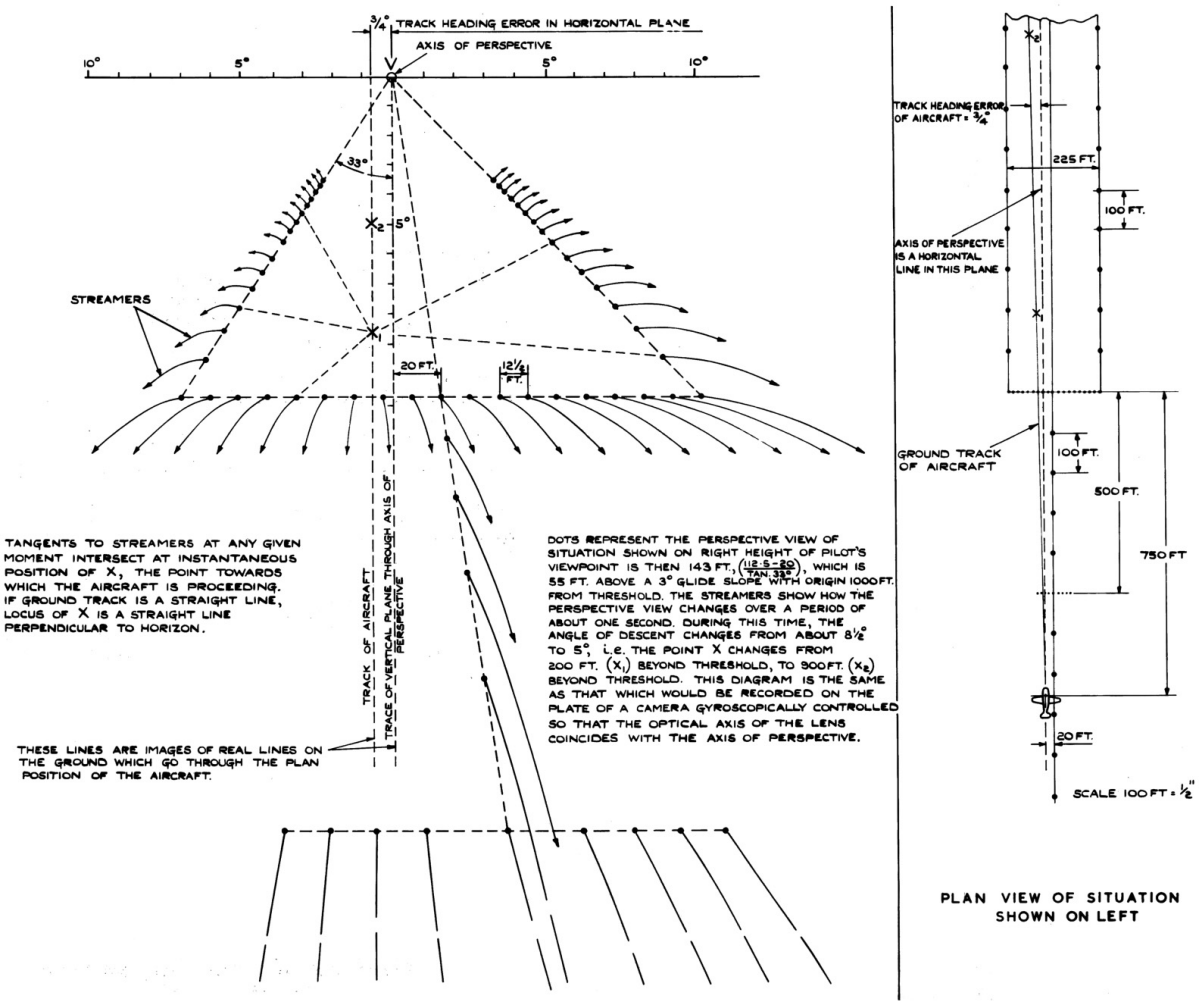
Illustration of the streamer pattern experienced during descent towards a runway (left). On the right, a top view of the depicted situation is shown. As is noted in the figure, the aircraft has a track heading error of 0.75° to the left of the center of the runway and changes its angle of descent from 8.5° to 5° during the depicted interval. Note that since this is a streamer pattern, each arrow depicts the retinal trajectory during an interval, not the instantaneous retinal velocity of fiducial points. Reproduced from Calvert et al. (1963). The same figure appears in Calvert (1955a, 1957a).

Based on these two insights, Calvert formulated the parafoveal^24^ streamer theory and first presented it in his 1950 article. The parafoveal streamer theory describes how visual judgments during the final phase of approach and landing are carried out. It states that below a certain height, e.g. 150 ft., the pilot derives his impressions of altitude and direction from two sources of visual information. The first of these sources is the “streamer pattern” (Figure 11), which refers to the paths traced out by the approach lights and other points in the environment and can be likened to what in modern terms is called flow lines (Kim & Turvey, 1999; Lee & Lishman, 1977; Wann & Swapp, 2000; or more precisely, path lines, Warren et al., 1991b) as well as to motion streak (Burr, 1980; Geisler, 1999; see Niehorster et al., 2010 for the role of motion streaks in the direction of self-motion perception). The second source of information is the “streamer velocity,” or the visual velocity of points in the environment.

Calvert (1950) expounds the significance of this theory by describing the geometry of the landing operation. He states that “if an observer is in motion in a straight line towards a point X in a pattern, then all other points in the vicinity will appear to move radially away from X. … The streamers all pass through the point X … and this gives the observer a good clue to where he is going” (p. 203).^25^ Next, Calvert recognizes that the pilot can use this information to control his self-motion and aim to the touchdown point by “manoeuvr[ing] the aircraft until the lights appear to stream past [the touchdown point] steadily and symmetrically” (p. 203; see Warren et al., 1991a for the importance of the directional structure of optic flow for the perception of self-motion direction). He explains that the pilot then notices “consciously or subconsciously the rate at which the streamer velocities increase […, which] tells him his rate of descent” (p. 203; c.f., looming, Regan & Beverley, 1978; and Lee, 1976; Lee et al., 2009). Finally, in an appendix to the paper, Calvert (1950) develops a mathematical description of the retinal velocity of points in the field of view that is suitable for any angle of approach.

Four years later, Calvert (1954) further discussed the visual guidance of aircraft during approach and landing. In this paper, he extends his treatment of the problem by describing how the motion in the visual field indicates deviation from a desired path, as well as the rate and acceleration with which the deviation changes: “the present paper will concentrate on the indications which tell us where we are going rather than where we are, that is, those which give the rate of turn and the rate of closing the desired path” (p. 234). Specifically, he states that the rate at which a desired path is closed is specified by the distance between the zero point of the streamer pattern and the aiming point (the track heading error). The rate of change of closure (the turn rate, or path curvature) is specified by the speed at which the zero point moves with respect to a fixed reference. It is of specific interest to note that Calvert (1954) envisions steering to aim toward a target as putting the zero point as close as possible to the target. Here, for the control of glide slope (angle of descent):

*If the aircraft is to descent towards [an aim point that he is currently overshooting], the pilot must operate the controls so as to initiate a rate of change of track heading downwards, i.e. he must give [the zero point] a velocity towards [the aim point]. As [the zero point] moves towards [the aim point], the downward velocity of [the aim point] decreases, and becomes zero when [the zero point] is on [the aim point]. The aircraft is then tracking towards [the aim point]. (p. 244)*

**Figure 12.**
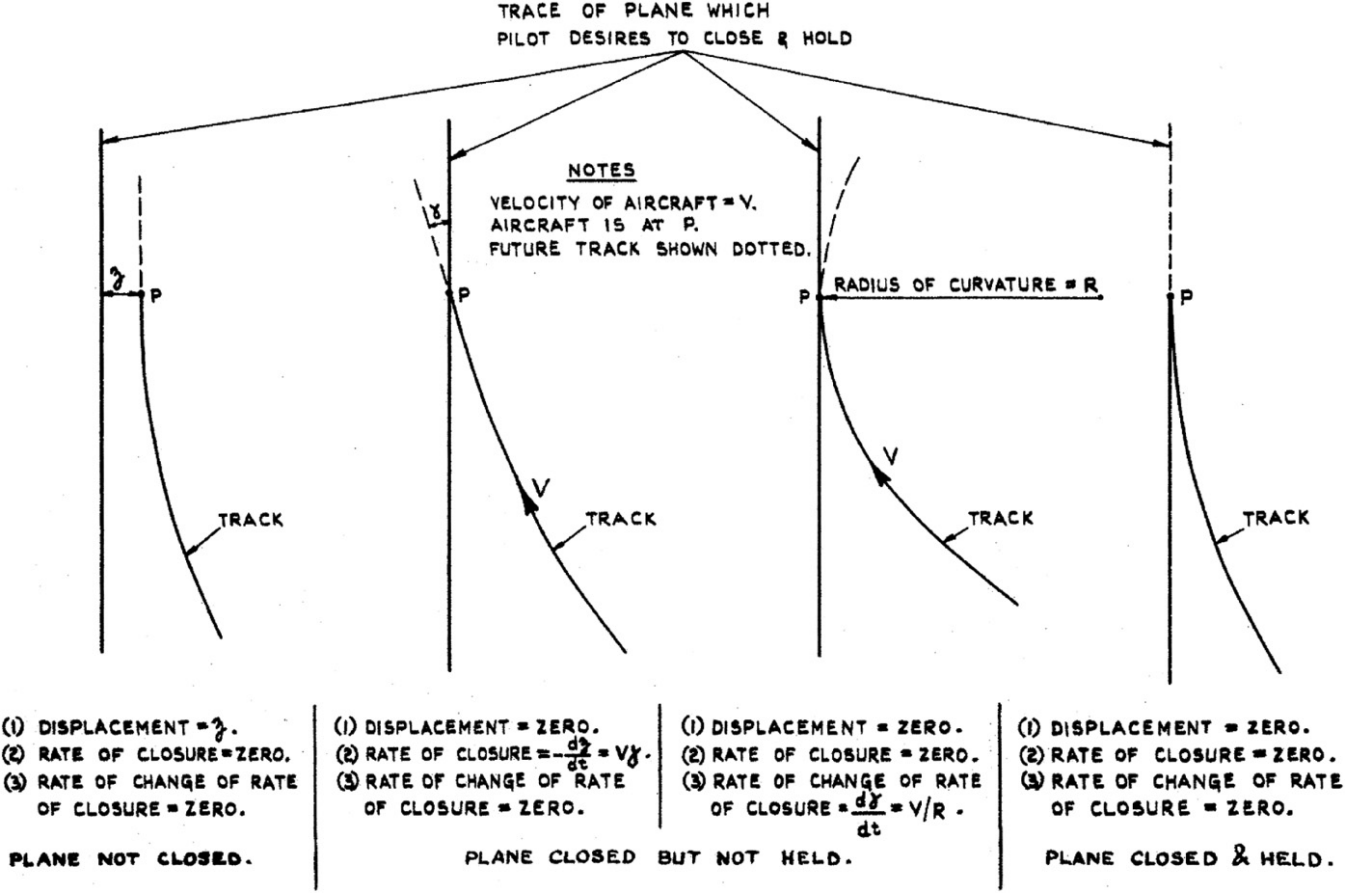
Illustration of the conditions to be met to turn into alignment with a desired path. Depicted are four situations, only one of which (the right) leads to successfully turning the vehicle into alignment with the path. For each situation, the track along which the aircraft traveled to its current position P is drawn. Furthermore, the future track determined by the instantaneous displacement, rate of closure, and change of rate of closure at point P is plotted as a dotted line. Reproduced from Calvert (1955b). The same figure appears in Calvert (1954, 1955a, 1957a).

Calvert (1954) devised specific control strategies that elaborate on the above. These strategies use the visual motion information to close a desired path such that at completion of the maneuver, the aircraft is aligned with the path and tracking along the path (see Figure 12). It should be noted here that the complete strategy for closing a path consist of more than bringing the zero point to an aim point, it also includes an appreciation of the importance of the turn rate, that is, the rate-of-change of the position of the zero point in the visual scene.

In a lecture in 1955 to the German Scientific Society for Aeronautics, Calvert (1955a) discussed some of the factors that affect the accuracy of judgments from the streamer pattern. As Grindley and Gibson also observed, he noted that eye movements disturb the pattern. Furthermore, as streamer velocity is inversely proportional to the distance of the object, he stated that resolvable detail in the foreground, such as provided by the crossbars of his approach lighting pattern, is needed (c.f., Li & Niehorster, 2014; Li & Warren, 2000). If only objects further away are available, “the pilot has only very slow and indefinite streamers from which to find [the zero point], and is like a fly on a windowpane until he gets very close to the runway” (Calvert, 1955a, p. 109). He held that the absence of resolvable detail in the foreground is also the reason why accurate approaches are hard to achieve when flying over featureless terrain such as the sea, desert and snowfields.

In 1957, Calvert further expanded on how displacement, track heading and track curvature—or “where [one] is at any moment, where [one] is going at that moment, and … where [one] will be going a few moments later” (Calvert, 1957a, p. 272)—are perceived by the pilot in the horizontal and vertical planes. As we have seen above when Calvert discussed “[giving the zero point] a velocity towards [the aim point]” (1954, p. 244), previously, he did not show an appreciation for the fact that a laminar motion is added to the visual motion pattern when the observer's orientation changes, such as when traveling along a curved path. He only stated that one should avoid making eye movements (Calvert, 1950, 1954) to avoid the addition of motion components which confuse the streamer pattern (Calvert, 1955a). However, when discussing track curvature, Calvert (1957a) now notes that curvature “is given by translatory [(laminar)] movement of the whole perspective picture relative to the [airplane's] framework, this motion being superimposed on the streamer flow produced by the forward motion”^26^ (p. 278). Calvert here recognizes that while the laminar component could confuse the streamer pattern's indications of travel direction, it is also of use as it provides a visual indication of the rate of turn. He appears to assume that the visual motion components due to forward motion and rotation are effortlessly decomposed and that self-rotations thus pose no problem for direction of self-motion perception (see, e.g., Warren, 2008; and Li et al., 2009 for discussions of this “rotation problem”).

In 1954, Calvert framed the visual guidance of aircraft landing as a temporal succession of perception and action (see quote above). On this subject, he now used an explicit control theoretic formulation, which engineers such as he would be well familiar with (see Calvert, 1956), when he noted that: “these three quantities [position, track heading and turn rate] appear as changes in the driver's visual environment, and there is feedback of information from this environment to the driver which modifies his behavior” (Calvert, 1957a, pp. 272–273). He thereby foreshadowed the perception/action loop model of visual guidance that Gibson would introduce a year later (Gibson, 1958; Warren, 1998). In fact, a statement similar in spirit is found ten years earlier when Calvert stated that “the process of learning [to fly an aeroplane] then consists in acquiring a chain of conditioned reflexes linking the movements of the controls with the movements of certain significant parts of the outside world” (Calvert, 1947c, p. 6).

Lastly, Calvert (1957a) expands on his previous treatment of altitude guidance by observing that the rate of approach of the ground is proportional to the distance of the zero point from the horizon. However, he concedes that altitude guidance based on visual motion information remains difficult, even in good viewing conditions, as he reckons that the speed of the visual motion pattern only becomes sufficient for adequate altitude guidance at altitudes as low as 100 feet (see Calvert & Sparke, 1957 for a more extensive discussion). As such, Calvert argues that little can be done in terms of approach pattern design to remedy the situation. However, as knowledge of altitude and the angle under which the ground is approached is crucial for altitude guidance of the aircraft and safe landing, he instead recommends (Calvert, 1957a, 1959) installing Visual Approach Slope Indicators (Sparke, 1958, 1960; see Baxter et al., 1960a, 1960b; Day et al., 1960 for tests of such systems) that signal whether the aircraft's angle of approach and altitude are appropriate.

At around this time, perhaps because approach lighting systems for precision approach runways were now mature in his view (Calvert, 1959), Calvert shifted attention to other topics. For instance, he designed a simplified approach lighting system for airfields that are only used in good visibility conditions (Calvert & Sparke, 1962), and he analyzed how visual and instrument manual landing systems are best combined with automatic landing systems to optimize the safety of air transport operations and operational throughput (Calvert, 1959; Calvert et al., 1963; Calvert & Sparke, 1957).

Furthermore, he became interested in collision avoidance, starting with a working paper to facilitate discussion of the problem at an ICAO Airworthiness Committee meeting (Calvert, 1958a). At the time, collision avoidance at sea and in the air was a fiercely debated topic of great interest given that there were 159 mid-air collisions in the period 1948–1957 in the United States alone (Calvert, 1960; see Sharpey-Schafer, 1955 and Wylie & Deacon, 1963 for ship collision statistics). Several mathematical treatments of the problem were developed (Calvert, 1958a; Morrel, 1961; Sadler, 1957),^27^ which led to the development of several systems for collision avoidance (Calvert, 1960, 1973; Hollingdale, 1961; Wylie, 1960), investigations of the human factors aspects of the problem (e.g., Broadbent, 1958) and discussion about changing the rules of procedure at sea (Calvert, 1969, 1971; Kemp, 1965; Kemp et al., 1965).

## Discussion

### Did Gibson Take the Concept of Optic Flow from Grindley?

Mollon (1997) has previously claimed that Gibson's discovery and description of optic flow is derivative of Grindley’s (1942) report. Mollon substantiates this claim by noting some similarities between the contents of Grindley's report and Gibson's work and by stating that Grindley had told others that his report was sent to Gibson. As Mollon (1997) also identified, the only reference Gibson makes to Grindley is in a footnote when discussing that the optical center of expansion is on the ground during the landing of an airplane: “G. C. Grindley has described this and some other aspects of the phenomenon in a brief report to the British Flying Personnel Research Committee. Whether a discussion of the phenomenon has been published could not be determined at the time of writing this report” (Gibson, 1947, p. 227).

Indeed, as discussed above in “The Motion Perspective Gradient” section, Gibson's formulation of the information about self-motion contained in retinal flow and its application in the landing of an airplane, started in 1942 with a summary of Grindley's report found among Gibson's notes (Gibson, 1942–1945). This confirms that Gibson received Grindley's report and studied it in detail. Furthermore, in his summary of Grindley's report, Gibson for the most part used Grindley's terms. One exception is Gibson's use of the term “expansion” to describe the retinal motion pattern, where Grindley (1942) used “spreading out” (c.f. the “Dauser (1939)” section above). However, up to what extent is Gibson's later work (Gibson, 1947 and onward) derivative of Grindley's report?

Here, it should be noted that Grindley's report was chiefly concerned with judging altitude from the retinal motion patterns, not with a discussion of how self-motion could be perceived from the retinal motion: “what the pilot has to do [… in landing an aeroplane] is to judge his absolute distance from the ground” (Grindley, 1942, p. 1, underline his). Only in one instance does Grindley mention that aspects of the retinal motion pattern are related to self-motion perception when he said that “[the pilot should] be able to infer his angle of descent from the position of the zero point” (p. 2). It thus appears that Grindley saw the zero point in the retinal motion pattern at the direction one is moving toward as an optical fact due to the laws of perspective, but otherwise did not attach significance to it.

Although Grindley's report might have made Gibson aware of some of the optical properties of the retinal motion available to an observer during self-motion, Gibson's treatment of its function for perception went far beyond Grindley's report. First, Gibson recognized that the retinal motion pattern experienced during landing is but one instance of the retinal motion patterns experienced during everyday locomotion, whether it be on foot, in a car or in an airplane. As such, most of Gibson's discussion of the self-motion information contained in motion perspective concerns locomotion parallel to the ground plane, which is the case generally experienced during our everyday life.

Second, Gibson recognized that the main role of the retinal motion experienced during locomotion is to inform the observer of his direction of self-motion, not his altitude. Indeed, both Grindley and Gibson realized that the retinal motion information specifying altitude is confounded with one's ground speed.

Last and most important, Grindley held that the velocity of individual points, instead of the retinal motion pattern as a whole, provides information for the perception of altitude. While this is in principle correct provided one knows one's groundspeed, it also illustrates that Grindley took an atomistic perspective and was not concerned with the information that can only be provided by the *pattern* of retinal motion. For as far as can be judged from his report, Grindley thus did not recognize the importance of the expanding retinal motion pattern in perceiving the direction of self-motion, nor did he inspire Gibson's ground theory in which retinal gradients such as that of decreasing speed with increasing distance provide the cornerstone of terrestrial space perception.^28^ It is important to stress here that observing and briefly describing an optical phenomenon as Grindley did should not be equated to Gibson's subsequent posing of the theory that higher-order properties of the world and oneself can be directly perceived from patterns in retinal stimulation. This radical proposal turned on their head the atomistic doctrines of perception that had at this point been established for decades. While it is thus interesting to consider that Gibson might never have discovered optic flow without a program for sharing information and research reports between the US and the UK during WWII, I posit that Gibson's published work is not derivative of Grindley’s (1942) report.

One other claim of Mollon (1997) deserves further attention. He states that “Grindley (1942) makes the point, that later turns up in *Perception of the Visual World* (Gibson, 1950), about a constant being added to all velocities if one tracks a point in the field” (Mollon, 1997, p. 861, italics his). Grindley, however, in his report only noted that during rotation, the retinal motion of points in the world does not depend on the distance of the points and that during simultaneous translation and rotation the optical motion of a point is a sum of the components due to translation and rotation. Grindley did not provide any further interpretation in terms of the effect of eye movements on the retinal motion pattern. Gibson's statement (Gibson, 1947, p. 225, see “The Motion Perspective Gradient” section) that eye movements add a constant velocity to all points in the field—and thus do not affect the motion perspective gradient—is therefore original. However, given Grindley's evident understanding of the retinal motion generated by self-motion and -rotation, it is likely that he would have immediately agreed with Gibson's assertion.

It is also of interest to note the similarities between Grindley's and Gibson's methods for constructing a selection test probing the ability of prospective pilots to make visual discriminations related to landing aircraft. Although Grindley's aim of making a test of visual velocity discrimination is different from Gibson's aim of testing perception of the aim point of a landing glide, the method that Grindley suggests for creating the test is very similar to that used by Gibson. Grindley notes that landing can be simulated with a contraption in which an inclined platform mounted on a moving trolley approaches the observer. This is in essence the same as Gibson's method of moving a camera along an inclined track toward a miniature airfield to produce a film simulating the view of the pilot during landing. Furthermore, Grindley realized, as did Gibson, that films taken from the viewpoint of the pilot might be useful in the instruction of cadets. There is no evidence available that allows a determination of up to what extent Grindley's suggestions discussed in this paragraph influenced Gibson's methods of selecting and training plots.

Last, we should wonder why Gibson did not cite Grindley's work in his later publications. This is perhaps because Grindley's report was classified as secret by the RAF and was only finally declassified in 1972 under the UK's 30-year rule. As such, referring to the report outside of a military document (which Gibson, 1947 was) would have been impossible. Indeed, even Gibson's 1942 internal-use only summary of Grindley's report did not identify the report's source and author (nor was the author of the abstract listed) because Grindley's report was given the American “confidential” classification (letter of Maj. Geldard to Lt Col. Flanagan, September 7, 1942; in Gibson, 1942–1945). Gibson (1957b) sheds further light on the problems that classified materials pose for scientific discussion, noting that publications which are classified “could be read, after delay and difficulty, but not kept and not referred to in print” (p. 130). He was quick to make sure the reader knew what he thought of this practice: “This is the sure and certain way of strangling scientific communication. … Interesting research … on optical information for flight, even today, is being carried out without permission to publish. It will become ingrown and provincial as a result” (p. 130).

### Gibson and Calvert

Calvert, not encumbered by knowledge of the contemporary theories of perception, developed theories and descriptions that in many aspects were much closer to Gibson's than Grindley's. Importantly, Calvert's parafoveal streamer theory was not atomistic because he held that the streamer pattern as a whole, not just its zero point, provides information about self-motion—this is clearly illustrated by the term parafoveal in its name and the quotes provided in the “E.S. Calvert” section above. Furthermore, Calvert's theory of how ego-position and ego-motion are perceived clearly demonstrates that his thinking never was atomistic even before he appreciated the streamer pattern: “Everyone who has the use of his eyes judges his position in space by noticing the pattern formed by the objects around him, and finds his way about the world by noticing how this pattern changes” (Calvert, 1947c, p. 6). Multiple other similarities can be noted between statements made by Gibson and Calvert, some of which I will briefly discuss here.

For instance, as already noted above, both Gibson (1958) and Calvert (1947c, 1954; 1957a, see the “E.S. Calvert” section above) couched their discussion of the visual guidance of aircraft in cybernetic terms such as feedback loops and discussed the interplay between perception and action. More specifically, both realized that a vehicle can be steered to a target by bringing the zero point to the target.

Furthermore, Calvert also appears to have appreciated Gibson's distinction between the retinal image and the potential optical stimulation available for an eye at a given point of observation (Gibson, 1957a, 1961). Calvert (1956) states that he would precede his terms (e.g., streamers) with “retinal” when referring to the retinal image instead of the “perspective image.” Strictly speaking, however, Owen’s (1990) statement that Calvert's perspective image refers to the same concept as Gibson's optic array is incorrect because Calvert's image is planar and yoked to the orientation of the aircraft. While this is appropriate for the practical purposes Calvert concerned himself with, Gibson's ambient optic array is unoriented and encompasses light incident from any direction (see Gibson, 1979, p. 186).

Perhaps the most curious among the similarities between Calvert and Gibson is that reminiscent of Gibson's ground theory, Calvert (1955a) also discussed how the ground plane and the objects on it provide structure to the visual world. He stated that “in addition to providing streamers, these objects provide a continuous framework of size and distance, which makes it very much easier for the pilot to recognize the visual situation for what it is” (p. 110). Calvert continued:

*A pattern which regularly repeats itself, as for instance grass and shrubs, or bricks in a wall, is particularly effective in giving the impression of a plane, and its inclination to the observer's line of sight. … The appearance of reality and solidity in a pattern due to the presence of objects of known transverse dimensions, repeated in a regular sequence longitudinally, is what we have come to call “texture”. … The pilot usually describes the effect of texture by saying that it helps him to “establish his ground plane”. Ground plane in this sense is an impression of height, combined with an impression of the inclination of the plane of the ground to the framework of the aircraft, i.e. it is a combined impression of height, pitch angle and bank angle. (pp. 110–111)*

Calvert gives here an explicit account of how an observer perceives the structure of the visual world and assigns primacy to the texture of the ground plane in enabling the observer to perceive both his ego-position and ego-orientation.

As noted above, Gibson once stated that “the problem of … distance perception is basically a problem of the perception of a continuous surface[:] if there were no surface, there would be no visual world” (Gibson, 1947, p. 185). Similar to this statement, Calvert (1955a) noted the unreality of a space devoid of texture:

*Many of you will, no doubt, have noticed that the perspective pictures in this paper [(e.g., Figure 11)] are unrealistic in that they do not give the impression that the space between the cut-off line and the horizon is the ground plane stretching away to infinity. Well, the picture seen by the pilot as the ground first looms up out of the fog has a good deal of the same unreality, and some of the pilot's difficulties [in low visibilities] undoubtedly spring from this (p. 110)*.

In earlier work, Calvert also discussed the importance of texture. Below, he demonstrates observation skills that are sufficiently keen to make him a good vision scientist:

*Texture gives the whole [approach lighting] pattern an appearance of reality when seen in fog, and makes recognition of the ground plane much more rapid. (One has only to look at the sky at night to realise that patterns consisting of pin-points of light do not appear to lie in any particular plane.) This is because we recognise the ground plane by seeing objects on it such as trees, shrubs, houses etc. whose real sizes we know. (*Calvert, 1949*, pp. 12*–*13)*

Calvert is shown here to also appreciate the point made by Gibson (1947) that the perceived distance of objects becomes indeterminate in the absence of the continuous background provided by a textured plane. He even provides the same example as Gibson (1947, p. 186) that objects seen against the sky do not have a determinate distance. From these quotes, it is clear that Calvert would certainly have agreed with Gibson's proposal of the ground plane as a framework for distance perception.

Nevertheless, we should wonder whether Calvert was an observer with a keen eye for the factors that provide for perception of the world, or whether he was simply influenced by Gibson. From the file that Gibson created on Calvert (Gibson, 1956), we learn that Calvert did not know of Gibson's work until early 1956, showing that the similarities noted above which are taken from work published in 1955 and earlier are examples of convergent evolution of thought. In February 1956, Gibson, who by that time was aware of Calvert's work, took advantage of the opportunity of being in Oxford to look up Calvert and compare ideas. Before Gibson and Calvert met, Calvert wrote to Gibson explaining why he thinks very few psychologists in the US or the UK know the work done by him:

*The reason … is that the “psychology” is treated rather as incidental to the engineering & operational aspects. In other words, our simple theories of texture & retinal streamers gave us such instant & startling success when applied in 1947/1948 that we treated them as simple truths obvious to everyone, & didn’t even bother to write them up to any greater extent than we needed for our immediate practical applications. (Feb. 16, 1956; in* Gibson, 1956 *)*

Calvert attached an reprint of Calvert (1954) with this letter and promised to bring with him a copy what likely was of Calvert (1955b)^29^ for their meeting. Gibson wrote to Dallenbach (October 11, 1956; in Gibson, 1956) that after a whole day with Calvert, he was satisfied that there were no fundamental disagreements in their mathematics (see also Gibson, 1957b).

The discussion between Gibson and Calvert must however have been difficult at times as their meeting prompted Calvert to write a letter to The American Journal of Psychology where Gibson et al.'s (1955) mathematical treatment of motion perspective had just been published, mainly commenting on the terminology used in that paper. Gibson was irked and surprised that Calvert’s (1956) note was published and enquired of the editor Dallenbach why it was accepted (October 11, 1956; in Gibson, 1956). Gibson found the first sentence of Calvert’s (1956) note (see below) “extremely snooty (besides being incorrect)” but promised to be polite in his reply to Calvert. In a reply to Gibson's letter, Dallenbach (October 13, 1956; in Gibson, 1956) explained that he accepted Calvert's note because it was provocative and would draw a reply, which would stimulate interest in the field and the journal. Dallenbach's motives were also political, however, as he explained to Gibson that “rejection would give some justification to [Calvert's] contention that American psychology was provincial and unwilling to consider ideas from abroad.”

Calvert (1956) opened his note by stating that Gibson’s (1955) article “puts forward the beginnings of a theory which has been carried much further in the United Kingdom—but by engineers and pilots, not by psychologists” (p. 476). He continued, “if the psychologists are going to join in this work [developing a theory of how visual judgments are made in motion], and it is highly desirable that they should, then I suggest that they begin by adopting the terminology which the engineers and pilots already use.” In his view, “the psychologists working in the field of visual perception seem to be so lax in [carefully defining their terms] that their writings have little meaning for the pilot or engineer.” Calvert then proceeded to critique the terminology of Gibson et al. (1955), and to inform psychologists of the importance of rate-of-change information in the achieving of stable control of a moving vehicle, something that he contended is well known to anyone who has worked on the problem of control. He ended by stating that, “if psychologists wish to make a useful contribution, they would be well advised to make themselves familiar with this [his] way of thinking about the visual control of aircraft, and to adopt in the future a less muddled terminology” (p. 479).

Gibson (1957b) was quick to fire his “broadside” (letter of J.J. Gibson to K.M. Dallenbach, December 3, 1956; in Gibson, 1956)^30^ in response to Calvert's “rather blunt advice” (Gibson, 1957b, p. 129). After noting that his theory has been in print longer than Calvert's, Gibson points out that while scientific terminology is established by a body of literature, Calvert curiously fails to even give reference to his own work when telling psychologists that they would be well advised to know about his way of thinking. In connection to this point, he lamented the restrictions put on the publishing of interesting research because some of the work is classified (see the end of the “Did Gibson Take the Concept of Optic Flow from Grindley?” section above). Gibson (1957b) then clearly outlines the logic behind his choice for the term motion perspective, although he also concedes his choice might have been unfortunate. Last, in a noteworthy paragraph, Gibson closes off his reply by explaining how scientific terminology evolves:

*Calvert is an engineer who wishes to prevent accidents. I am a psychologist who wishes to understand visually guided locomotion. When he argues that the proper meanings of terms like parallax, perspective, orientation, and texture are to be found in any technical dictionary and that any effort to change these meanings only muddles thinking and confuses the reader, I have to answer that new concepts demand changes in the meanings of old terms. A good theory justifies a great deal of linguistic inconvenience; and in the long run a good theory will prevent more accidents than a muddled theory.^31^ (p. 131, emphasis his)*

Gibson's reply came too late for Calvert's next publication (Calvert, 1957a), however, where Calvert made similar comments about the theorizing of psychologists.

Vexed as Gibson may have been at Calvert's note, there are signs that it did have some effect on his thinking and use of terminology. As Owen (1990) also notes, Calvert's critique of the term *motion perspective*, a term Gibson (1957b) already concedes might have been unfortunate, may have driven Gibson to later note: “A better term would be flow perspective, or streaming perspective” (Gibson, 1979, p. 227). Moreover, the cybernetic turn that Warren (1998) identifies in Gibson’s (1958) thinking about the visual guidance of locomotion might have found some inspiration in Calvert's (1954, 1956) description of the necessity of rate-of-change information for the stable control of a vehicle.^32^

Overall, in Calvert we find a keen observer with a sharp eye for the optical facts that matter in perceiving the world and one's position in it and motion through it. As Gibson (1957b) also observes, Calvert's theories are largely the result of introspective efforts that left him convinced about what information a pilot needs in completing a landing as well as which particular judgments the pilot makes during the process. Perhaps because the results of these efforts bore him great fruit, he presented his convictions as facts instead of as hypotheses. Nevertheless, Calvert independently developed theories that echo many aspects of Gibson's work. Sadly, as Calvert was already seen writing to Gibson above, he seemed to have little interest in developing his convictions into full-blown theories and presenting these in publications. Instead, as can be appreciated from my discussion of Calvert's work in this section and the “E.S. Calvert” section above, he scattered his conceptions throughout his publications and many reports, each time lifting different tips of the veil only for as far as necessary to support the particular point he set out to make.

In closing, it is interesting to observe from Calvert's story that a deep thinker and keen observer not encumbered by contemporary psychological thought can sometimes penetrate much closer toward a fruitful new doctrine of perception than the perceptual psychologists that were his contemporaries. More generally, looking across the many contributions made on the topic before Gibson, one is struck by the difficulty of grasping a new idea fully when it first comes into view. For instance, Grindley (1942) was hampered by his conceptual framework of clues (for distance perception) and Tschermak-Seysenegg (1939) could not look beyond distance perception when thinking about the cue of motion perspective. While the discussion provided by both indicates they likely sensed there was more to this phenomenon of optic flow (Grindley even drew out the pattern of velocities), neither was quite able to grasp it. When it came to precisely expressing what they were thinking, they could only do so in terms of optical velocity as a cue to the distance of a point in space, which was the conventional reductive, point-wise, analysis of their time. For Gibson to develop the concept of optic flow, he had to first step outside the doctrines of his time and develop the idea of a “higher-order variable” (Warren, 2005, pp. 342‒344), which then allowed him to think of a pattern (such as a flow or a gradient) as stimulus information. Comparing the writing of perceptual psychologists like Grindley and Tschermak-Seysenegg to those of a relative outsider like their contemporary Calvert leaves me to wonder how much fruitful new insight is currently not developed only because our vision and thinking are constrained by the conventions, theories, and analogies that currently hold sway among perceptual psychologists.

### On Terminology: “Heading”

The reader might have noticed that the now ubiquitous term “heading,” referring to one's instantaneous direction of self-motion, is curiously absent from the above discussion, even though the term is usually ascribed to Gibson (1950). In fact, to the best of my knowledge, the term “heading” was introduced into common usage in the literature on self-motion perception by William Warren in 1988 (Warren et al., 1988). Bill Warren recounted that he coined “heading” when he was looking for a succinct term to designate “direction of self-motion” and other such phrases that were in common use at the time (personal communication, September 6, 2013). The term heading has also been used earlier by Warren (1976),^33^ who used it as a short form of “ego-heading,” in turn a derivative of his term “ego-motion.” Calvert (e.g., 1954) sporadically refers to the direction of self-motion as heading, but almost always uses the more precise term “track heading.” Gibson usually used phrases such as “direction of locomotion” and is seen to use “course” in his early notes (Gibson, 1942–1945), but to the best of my knowledge never used the term heading. A more thorough investigation of how the concept of heading was developed and studied was sadly hampered by an inability to acquire a significant portion of the relevant literature^34^ and will thus not be pursued further here.

As some authors have pointed out (e.g., Beall & Loomis, 1996; Cutting et al., 1992; Warren, 1990), the term heading can cause some confusion for it has more than one meaning both in its colloquial and scientific usage. In aviation and marine vehicular control, the term heading refers to the orientation of the vehicle, which coincides with the axis of thrust, but not necessarily with the direction of locomotion as these vessels travel through non-solid mediums such as water or air. Even for terrestrial locomotion on its solid substratum, orientation of the body axis does not necessarily coincide with the direction of locomotion, such as when walking crab-like or when a vehicle skids over a slippery road. Warren et al. (1988), however, used the term heading in its other definition, which would be referred to as “course” in the control literature. Warren et al.'s (1988) usage was based on colloquial phrases such as “where you are heading?” and “I’m heading to the left of the obstacle” and is also supported by the dictionary definition of heading (Warren, personal communication, September 6, 2013). He decided that its technical meaning was sufficiently arcane and thus opted to use heading in its colloquial meaning (Warren, personal communication, February 19, 2021). For the interested reader, Owen (1990) provides a detailed discussion of other terms used in the field of self-motion perception and control.

## Notes

1. As Warren (1982) notes, science fiction writers such as Arthur Clarke (Clarke, 1969/2000, ch. 41) have described the flow of the retinal image during self-motion. As Gibson (1947, p. 228) notes, Langewiesche's classic text on flying a plane also described the use of the expanding flow in landing an airplane (Langewiesche, 1944, p. 284‒286, 304), although most of the description of this cue is in chapter 15, not 16 as cited by Gibson.

2. That is, “optic flow.”

3. In other words, the visual angle subtended by a sphere that is approached will grow while simultaneously you can actually only see a smaller part of it.

4. It is interesting to note here that, as Boynton (1974) and Cutting (1986) discuss, in some sense Gibson's ecological optics is not so much a new theory of optics, but a return to classical optics as expounded by Euclid and other classical and medieval scholars.

5. It is noteworthy here that Helmholtz mentions the effect of rotation of head and body on the optical motions, but not the effect of eye rotations. As Rogers (2020, 2021) points out, Helmholtz, like later Gibson, considered the retinal image of little importance for perception and instead described stimulation in terms of the celestial sphere—a concept similar to Gibson's optic array.

6. Translator's note: while the German “Scheinbewegung” is better translated as “illusory motion”, in this article Tschermak-Seysenegg used illusory motion as meaning apparent motion (it is illusory in the sense that the world did not actually move).

7. Footnote in Dauser (1939): *Everything that applies to the perception of objective rest also applies to the case of objective movement. Only the description for this case is more complicated.*

8. With “different mathematical modes of transformation and motion,” Gibson (1957a) referred to the perspective, non-perspective, and disjunctive motions he discussed previously in his article.

9. Note that in a section titled “Linear responses to a rotating disc” (pp. 140‒141) he does describe how the outward motion perceived when rotating a disc containing a spiral pattern is consistent with translation toward the center of the disc.

10. Zangwill (1977) states that Grindley became a University Lecturer in 1936, not 1933 as recorded by Bartlett (1937). However, Bartlett (1937) is the source more likely to be correct.

11. For a further overview of Grindley's life and his work after the war, one can refer to his obituary by Zangwill (1977).

12. These dates do not match the dates in the account given by Mollon (1997). However, Mollon's account is third hand, based on notes prepared by Bernard Babington Smith in 1978, based on a conversation with Grindley in 1959. The multiple intervening 20-year periods might have allowed some mistakes to slip in.

13. As per Whittingham (1939), several other members of the Cambridge Psychological Laboratory were put forward to study other topics: Mr. E.G. Chambers on the preselection of flying personnel; Mr. G.C. Drew on fatigue in aircraft crews; and Mr. K.J.W. Craik on the perception of relative (depth) position of objects. The numbers of topics studied as well as the number of Cantabrigians involved in the studies would increase significantly over the next few years.

14. Grindley has also produced reports on intercommunication in flying training aircraft (FPRC 85); the accuracy of reports made after reconnaissance flights (FPRC 120) and fatigue and stress in aircrew (FPRC 121). Later, he worked on the selection of air traffic controllers (FPRC 423T, 612, and 612A).

15. This might have been because the standard velocity was always approached from below.

16. Edwin Garrigues Boring, the reviewer of the paper and editor of *The American Journal of Psychology* in which the paper was published, begged to differ. When he accepted the paper, he wrote in the enclosed letter: “I love your paper about how Lewin drives a car!” (Gibson, 2002, p. 27).

17. This anonymous article was written by Gibson. The work of other units is discussed in other reports in the same series as Gibson (1947). See Flanagan (1947) for an overview of the available reports.

18. Parts of Gibson (1947, chapter 9) are reprinted in (Gibson, 1982) as chapters 1.1 and 2.2. Chapter 1.1 discusses depth perception from retinal gradients, while chapter 2.2, a later section of Gibson (1947, chapter 9), discusses the visual perception of self-motion from the motion perspective gradient.

19. Note that while Gibson uses the term “retinal flow” in this passage, he does not do so consistently in this text. He uses other terms, such as “motion perspective” and “flowing deformation”, to refer to the retinal flow, and mostly uses the word flow as a verb, for example, “The fact to be especially noted is that the retinal image over the whole retina, the image representing the entire visual field, may undergo this kind of motion, i.e., may flow at different rates in different parts” (p. 220).

20. Note, however, that the addition of a constant causes the location of the center of expansion to move, because of which it can no longer be used as information about the direction one is moving toward (see Gibson, 1950, chap 7; Regan & Beverley, 1982). This is known as the rotation problem and much work has been done on how the direction of self-motion is perceived despite the presence of eye movements. See Warren (2008) for a review.

21. Koenderink and van Doorn (1981) note that the term “focus of expansion” is technically a misnomer: the divergence at this point is actually zero. The point of maximum diverge generally lies elsewhere in the field. For instance, for translation parallel to a ground plane it lies on the floor at a distance of one eye-height in front of the observer (see also Koenderink, 1986). Gibson (1979) later used the term “focus of radial outflow”.

22. The following anecdote about how this collaboration came about is provided by Harold A. Sedgwick, a graduate student of Gibson who got to know Frank Rosenblatt in the 1965-66 academic year at Cornell when he took two courses with him as an undergraduate. Sedgwick later worked for Rosenblatt as a research assistant and lived for three years in the attic of Rosenblatt's farmhouse near Ithaca. As there was no evident connection between Gibson and Rosenblatt, Sedgwick asked how Rosenblatt, who was a doctoral student at the time, got involved in Gibson et al. (1955). Sedgwick recalls that the gist of Rosenblatt's reply “was that he happened to be walking by the open door of a classroom and saw Gibson and Olum working on a problem at the blackboard; he asked what they were doing, and they told him they were trying to work out the math for … motion perspective. In Frank's account, he did the math for them and went on his way. As I remember it, Frank made the encounter sound quite casual” (personal communication, August 11, 2013).

23. Calvert (1947b, 1947c, 1948) was the first to recognize that lateral deviation and bank (roll around the aircraft's axis) have the same rotating effect on the projection of a line on the ground. Any center line by itself thus provides ambiguous information about airplane state, which can be disambiguated by lines perpendicular to the centerline such as the horizon or the crossbars of the Calvert system (this is discussed in most detail in Calvert, 1949). With Calvert's (1947b, 1948) analysis of what he called the “perspective angle,” he was the first to describe what is now called (optical) splay angle (Warren, 1982): when parallel to the ground, one's lateral deviation from a line is optically specified by the angle between the retinal projection of the line and a vertical retinal reference (see, e.g., Beall & Loomis, 1996; Li & Chen, 2010).

24. It should be noted that Calvert's use of the term “parafoveal vision” refers to what is now commonly called peripheral vision: “the parafovea is, of course, what is commonly known as the ‘corner’ or ‘tail’ of the eye” (Calvert, 1950, p. 203).

25. Although Calvert uses the term “point X” to refer to the optically static point at the direction where one is going, for clarity I will replace it with the term “zero point” in the rest of this discussion.

26. Although Calvert is seen using the term “flow” here, it should be noted that he did not do so in any of his earlier work. The use of this term was possibly inspired by reading some of Gibson's work or a meeting with Gibson in 1956 (see the “Gibson and Calvert” section below).

27. In their analyses, all these papers present the fact that a moving object that maintains a constant bearing is on a collision course with the observer. Although this criterion has been subject of the lore of the sea for centuries (Morrel, 1961) and thus did not originate from these authors, it is worthwhile to note that these mathematical tracts significantly predate Chapman’s (1968) paper on catching a baseball that is frequently mentioned as an early source in the moving target acquisition literature. Moreover, see Gerathewohl et al. (1970) for a good summary of Calvert’s (1958a) work.

28. Note that it is not known whether Gibson's ground theory and his discovery of gradients find their origin in his realization of the gradient of retinal motion during locomotion, or initially were independent developments.

29. Calvert's letter mentions “Report No. EL1468,” but he likely meant EL1486 (i.e., Calvert, 1955b).

30. Gibson was a good letter writer who would frequently make sure to provide some entertainment to the recipient. Dallenbach had sent over some reprints he thought were of interest. Gibson took the occasion of this letter as an opportunity to thank him, replying, “Thanks for the offprints. I was especially interested in the ones about theory. Wouldn’t you agree that the theory of theory is all right but the theory of the theory of theory is going too far?”

31. Note by author: c.f. Kurt Lewin's “there is nothing as practical as a good theory” (Lewin, 1943).

32. It is interesting to note that authors such as Reed (1988) attribute Gibson's cybernetic turn to a meeting with Gordon Walls. Gibson reading Calvert (1954), an annotated version of which is found among Gibson's files (Gibson, 1956), may however also have had an influence.

33. R. Warren (1976) presents the first empirical test of Gibson's hypothesis that the optical flow pattern available during locomotion over the ground plane provides an adequate basis for the perception of self-motion and its direction. Rik Warren recounted (personal communication, September 15, 2013, December 27, 2020 and February 20, 2021) that although he was a doctoral student with Gibson as his committee chair, the idea to examine heading perception from optical flow was not inspired by Gibson (although he immediately encouraged it), but was instead hatched during a walking conversation in 1972 at the Gibsons’ Ithaca Airport Laboratory with another member of his committee, Gordon Wilcox, a mathematical psychologist. While having his head turned to Wilcox and thus 90 degrees from his direction of travel, Warren mentioned that he could perceive his heading. When asked by Wilcox about how he knew where he was heading, Warren referred to Gibson’s (1950) diagram of lateral optic flow. Upon being pressed by Wilcox, Warren realized that Gibson's proposal, no matter how compelling the diagram is, was only a perceptual hypothesis, and one that was begging to be tested.

34. For reference, there are several technical reports dealing with self-motion perception that do not appear to have been given much attention in the scientific literature (besides some mentioned by Warren et al., 1988). These at least include Carel (1961), Gold and Hyman (1968), Kaufman (1968), Palmer (1968), Naish (1971), and Acree (1981).

35.
*Alles was für die Wahrnehmung von objektiv Ruhendem gilt, ist auch für den Fall des objektiv Bewegten giltig. Nur ist die Darstellung dafür komplizierter.*

## Appendix A

This appendix contains the original German quotes that appeared in translated version in The Germans section.

Tschermak-Seysenegg (1939)

p. 456:
*In dem speziellen Fall von Zufahren auf eine Schar von Objekten (oder Wegfahren von einer solchen!), die nicht geradlinig in der Bewegungsrichtung selbst gelegen sind, sondern sich rechts und links davon befinden, teilt sich die Schar in nach rechts und nach links mit verschiedener Geschwindigkeit abwandernde Einzeleindrücke. Dies ist speziell beim Geradeaussehen auf die beiden Fahrseiten vom Steuersitze eines Autos oder Flugzeuges aus (bei geringer Flughöhe!) oder vom Bug eines Schiffes aus der Fall; das Umgekehrte gilt beim blick vom Heck eines Schiffes oder vom Schlußfenster eines Zuges (Aussichtswagen) aus. In all diesen Fällen sind es Horizontalparallaxen, welche für den Entfernungseindruck in Betracht kommen […].*

p. 457:
*Einer solchen, ,statischen” Betrachtungsweise fehlt der unmittelbar zwingende Tiefeneindruck, wie er die Scheinbewegung bzw. die Kineoskopie begleitet!*

p. 467:
*Besondere Beachtung und Wertung verdient die kineoskopische Parallaktoskopie für gewisse Flugoperationen, so besonders beim Übergang zum Landen, also bereits in geringer Flughöhe, wobei emporragende Objekte (Türme, Schornsteine, Baume, Telegraphenstangen, Semaphore, Leitungsmasten, Häuser u.a.) eine deutliche horizontale oder speziell vertikale Bewegungsparallaxe gegenüber der Bodenflache erkennen lassen und dadurch eine relative Tiefenlokalisation bzw. Höhenschatzung, aber auch eine absolute Entfernungsbeurteilung gestatten. Aktive Kopf-Körperbewegungen werden dabei im Flugzeuge höchstens nebenbei zu Hilfe genommen werden.*

Dauser (1939)

pp. 5‒6:
*Wenn die Bilddeformation durch Bewegung des Körpers (Kopfes, Auges) vor sich geht, und nicht durch Formveränderung des Objekt, was sehen wir dann eigentlich? Sehen wir die Objekte und besonders ihre Formen, Konturen unverändert also unbewegt, weiterhin „objektiv“ wie sie sind? oder, wenn wir die Deformationsbewegung sehen, ist dieses Sehen bereits eine Wahrnehmungstäuschung? […] Denn wenn z. B. ich mich im Zimmer geradlinig bewege treten perspektivische Verschiebungen ein; diese könnten sowohl als Bewegungen der Objekte gesehen werden, wie auch durch Umstimmungen retinaler Ortswerte nicht gesehen werden.*

p. 16:
*[…] unser normales Wahrnehmen enthalt die perspektivische Verschiebung als Scheinbewegung der festen Objekte. Scheinwahrnehmen von Bewegungen ist also der Normalfall; aber — wir beobachten sie nicht, wir nehmen sie nicht ernst. Der Begriff der optischen Täuschung eignet sich nicht zur Bezeichnung dieses Sachverhaltes. Wir sehen etwas anders als es ist, lassen uns dadurch aber nicht täuschen.*

*Aber man muß noch weiter gehen. Wenn wir die perspektivischen Verschiebungen überhaupt nicht wahrnähmen, würden wir auch unsere Ortsveränderung im Raume der Objekte nicht wahrnehmen. Wenn wir andererseits die perspektivische Verschiebung in der Wahrnehmung ernst nähmen, wurden wir kein ruhendes Objekt als solches mehr erkennen. Allein die Wahrnehmung der perspektivischen Verschiebung zusammen mit dem Nichtbeachten und Nicht-Ernstnehmen dieser Bewegungen vermag die Wahrnehmung einer ruhenden Umwelt, in der ich mich bewege zu konstituieren.^35^ Die Scheinbewegung ist also konstitutiv für die Wahrnehmung einer festen Umwelt in der der Wahrnehmende sich bewegt.*
